# Amphipod family distributions around Iceland

**DOI:** 10.3897/zookeys.731.19854

**Published:** 2018-01-23

**Authors:** Saskia Brix, Anne-Nina Lorz, Anna M. Jazdzewska, Lauren Hughes, Anne Helene S. Tandberg, Krzysztof Pabis, Bente Stransky, Traudl Krapp-Schickel, Jean Claude Sorbe, Ed Hendrycks, Wim Vader, Inmaculada Frutos, Tammy Horton, Krzysztof Jazdzewski, Rachael Peart, Jan Beermann, Charles Oliver Coleman, Lene Buhl-Mortensen, Laure Corbari, Charlotte Havermans, Ramiro Tato, Anali Jimenez Campean

**Affiliations:** 1 Senckenberg am Meer, German Centre for Marine Biodiversity Research (DZMB), c/o Biocenter Grindel, CeNak, Zoological Museum Hamburg, Martin-Luther-King-Platz 3, 20146 Hamburg; 2 University of Hamburg, Biocenter Grindel, CeNak, Zoological Museum Hamburg, Martin-Luther-King-Platz 3, 20146 Hamburg; 3 Laboratory of Polar Biology and Oceanobiology, Department of Invertebrate Zoology and Hydrobiology, Faculty of Biology and Environmental Protection, University of Lodz, 12/16 Banacha st., 90-237 Lodz, Poland; 4 Natural History Museum, London, Cromwell Road, South Kensington, United Kingdom; 5 Tromso Museum, University of Tromso, 9037 Tromso, Norway; 6 Forschungsmuseum A. Koenig, Adenauerallee 160, 53113 Bonn; 7 Station Biologique, 2 rue Jolyet, 33120 Arcachon, France; 8 Canadian Museum of Nature, Ottawa, Canada; 9 National Oceanography Centre, Southampton, United Kingdom; 10 Coasts and Oceans, National Institute of Water and Atmospheric Research, 301 Evans Bay Pd, Greta Point, Wellington, 6021, New Zealand; 11 Marine Zoology, Bremen Marine Ecology (BreMarE), University of Bremen, PO Box 330440, 28334 Bremen, Germany; 12 Alfred Wegener Institute Helmholtz Centre for Polar and Marine Research, Am Handelshafen 12, 27570 Bremerhaven; 13 Helmholtz Institute for Functional Marine Biodiversity, Oldenburg; 14 Museum fur Naturkunde, Leibniz-Institut fur Evolutions- und Biodiversitatsforschung, Invalidenstra?e 43, 10115 Berlin; 15 Institute of Marine Research, PB 1870 Nordnes, N-5817 Bergen, Norway; 16 Museum national d'Histoire naturelle, Institut de Systematique, Evolution, Biodiversite ISYEB - UMR 7205 -CNRS, MNHN, UPMC, EPHE, 57 rue Cuvier, CP 26, F-75005, Paris, France; 17 Estacion de Bioloxia Marina da Grana. Universidade de Santiago de Compostela, Rua da Ribeira 1-4. A Grana. CP-15590. Ferrol. Galicia (Espana); 18 Laboratorio de Bentos Marino, Instituto del Mar del Peru, Esquina Gamarra y General Valle, S/N Chucuito, Callao, Peru; 19 University Museum of Bergen, PO box 7800, NO 5020 Bergen, Norway

**Keywords:** Amphipoda, benthos, deep sea, distribution, Greenland-Iceland-Faroe Ridge, subarctic, taxonomy

## Abstract

Amphipod crustaceans were collected at all 55 stations sampled with an epibenthic sledge during two IceAGE expeditions (Icelandic marine Animals: Genetics and Ecology) in 2011 and 2013. In total, 34 amphipod families and three superfamilies were recorded in the samples. Distribution maps are presented for each taxon along with a summary of the regional taxonomy for the group. Statistical analyses based on presence/absence data revealed a pattern of family distributions that correlated with sampling depth. Clustering according to the geographic location of the stations (northernmost North Atlantic Sea and Arctic Ocean) can also be observed. IceAGE data for the Amphilochidae and Oedicerotidae were analysed on species level; in case of the Amphilochidae they were compared to the findings from a previous Icelandic benthic survey, BIOICE (Benthic Invertebrates of Icelandic waters), which also identified a high abundance of amphipod fauna.

## Introduction

The international IceAGE project (Icelandic marine Animals: Genetics and Ecology) focuses on the climatic sensitive region at the northernmost part of the North Atlantic and the Nordic Seas (Greenland, Iceland and Nordic Seas reaching to the North Sea). The study area is characterised by a steep temperature gradient (< -0.9 °C to 14 °C) as well as several shallow (<800 m) submarine ridges which define distinct deep marine basins and host cold-water coral reefs along their slopes (Buhl-Mortensen et al. 2015a, b). Previous studies of benthic invertebrates in the North Atlantic and Nordic Seas including the BIOICE (Benthic Invertebrates of Icelandic waters: 1991-2004) and IceAGE (since 2011: see Brix et al. 2014a) projects have shown that within the abundant peracarid crustacean fauna, more than 50% of the species are new to science (Blazewicz-Paszkowycz et al. 2014). These projects have identified both broadly and narrowly distributed species in the region (e.g., Weisshappel 2000, 2001, Dauvin et al. 2012), where geographically restricted species are distributed to either north or south of Iceland (Svavarsson 1997, Brix and Svavarsson 2010). With cryptic species and species complexes a reoccurring theme for peracarid crustaceans, and particularly for amphipods (Havermans et al. 2013), there can be a significant underestimation of regional biodiversity (Just and Wilson 2004). Previous studies have indicated that integrative taxonomic approaches better allow for robust and transparent species delineation (Sites and Marshall 2004, Dayrat 2005, Leache et al. 2009, Padial et al. 2010). In order to best capture the diversity, distribution range and dynamic assemblage of the amphipod crustaceans, the first two IceAGE expeditions in 2011 (M85-3) and 2013 (POS456) expanded upon the traditional sampling and preservation methods from previous studies to incorporate molecular approaches.

Despite amphipods being the most common peracarid crustacean order within the IceAGE samples, prior to this study, amphipod crustaceans were underrepresented in project research outputs. The lack of scientific focus on this group was largely due to the large amounts of time and specialised expertise required to process the volume of material. At the beginning of the project, more than 66,000 amphipod specimens had been collected and were available for further identification (DZMB database, unpublished data).

Identifying amphipods is a complex task and owing to the "taxonomic impediment" the number of amphipod experts worldwide is in decline (Coleman 2015). Taxonomy is the fundamental science for understanding and assessment of biodiversity. All ecological and modelling analyses rely on accurate taxonomic information. Considering the known threats to biodiversity, new knowledge of existing species and the discovery of undescribed species from extant collections are urgently required. To overcome this impediment, the IceAGE team initiated several identification workshops. These workshops, run by senior experts, aimed to train early career researchers and to improve taxonomic knowledge of the amphipods around Iceland.

Here the results from two such workshops are presented. These results show the distribution patterns for amphipod families identified from IceAGE samples. For two abundant families, Amphilochidae and Oedicerotidae, species level identification is also presented.

## Materials and methods

**Figure 1.**
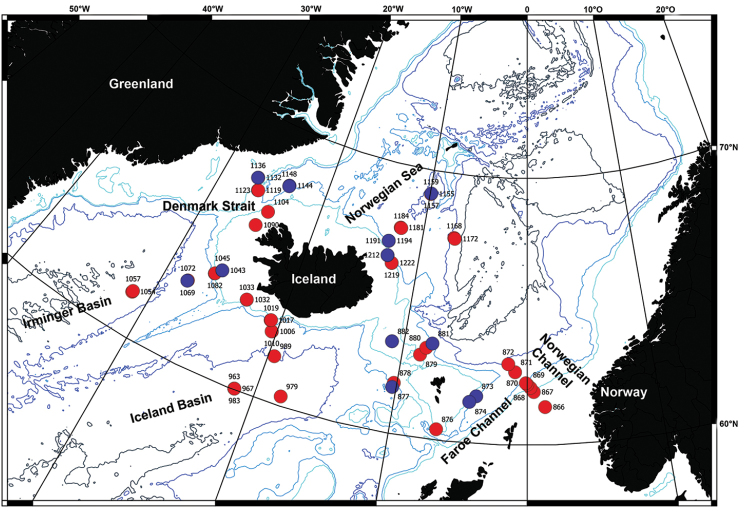
Map of all IceAGE EBS stations where amphipods have been found. Red: stations with amphipods determined; Blue: stations where amphipods were not further determined (873, 874, 877, 881, 882, 1043, 1045, 1069, 1136, 1144, 1148, 1191, 1209, 1212, 1157).

The IceAGE project and the expeditions were initiated and coordinated by Senckenberg am Meer (http://www.iceage-project.org), part of the Senckenberg Forschungsinstitut und Naturmuseum that serves to link scientists to samples collected by German research vessels and to make this material available. All sorting was handled according to Riehl et al. (2014) using an undisturbed cooling chain protocol. Following the fieldwork process and rough sorting of material to coarse identification levels, material from the IceAGE expeditions were housed in the Senckenberg "Meteor archives" (http://www.material-archiv.de/en/home.html). The IceAGE sampling protocol minimises mechanical nd physiological stress to specimens during the on-board rough sorting process. This protocol assists in preserving the integrity of the specimens for both morphological and molecular analyses (Riehl et al. 2014). In terms of expedition protocol, the sampling included six depth transects (1: Norwegian Channel, 2: Iceland-Faroe Ridge, 3: Iceland Basin, 4: Irminger Basin, 5: Denmark Strait, 6: Norwegian Sea) between 150 and 2850 m (Figure 1), where samples were collected using epibenthic sleds.

During both identification workshops, sample processing concentrated on amphipods collected with the epibenthic sledge (EBS). It should be noted that three types of gear were used during the IceAGE expeditions: RP sled (Rothlisberg and Pearcy 1977), Brenke sled (Brenke 2005) and C-EBS (Brandt et al 2013). Within these samples, identification was concentrated on those samples which were preserved in 96% ethanol to enable genetic work (see Jazdzewska et al. 2018). Only samples that would provide representatives of the most transects from the IceAGE station grid (Figure 1, red dots). As a result of this strategic sorting approach, a total of 21,658 specimens were identified to family level or lower.

For the families Amphilochidae and Oedicerotidae, all identified specimens have been registered in the permanent zoological collection at either Senckenberg (Frankfurt), the Naturkundemuseum Berlin or the Zoological Museum Hamburg (ZMH), Centrum fur Naturkunde (CeNak). All specimens selected as molecular vouchers (Jazdzewska et al. 2018) will be registered in the ZMH. At the time of manuscript preparation, the higher classification of the Amphipoda was in a state of reassessment (Lowry and Myers 2017). Our paper follows a conservative classification to allow preparation of the material in line with the World Amphipoda Database as of May 2017 (Horton et al. 2017).

Due to the "expert-bias" of participants at our two workshops and the incomplete sorting at family level, small families often received a more detailed treatment, while some larger taxa such as the Lysianassoidea or Phoxocephalidae were dealt with quite cursorily. Families that are known to be very abundant in Icelandic waters, including e.g., the Ampeliscidae (Dauvin et al. 2012) were underrepresented in our samples as we focused on ethanol-fixed samples collected by an epibenthic sledge, which does not adequately sample the Ampeliscidae. The approach to processing this extensive amphipod collection did not allow enumerating the species in every family. Despite these shortcomings, our results can provide a preliminary insight into the Amphipoda collected during the IceAGE expeditions.

Certain findings of singletons or rarer taxa are important for particular families, i.e., the Sicafodiidae (Campean and Coleman 2017) which is the first record of the family in the northern hemisphere. Here, singletons were excluded from the analyses to reduce "noise". Distribution maps are provided for the families (or superfamilies) recovered (excluding singletons) with a brief description indicating its significance in the region.

### Data analysis

Distribution maps were created for the amphipod families, one superfamily (Lysianassoidea) and one infraorder (Corophiida) occurring at more than two stations using the freeware QGIS, and were assembled using Photoshop CS6. Multivariate analyses were performed on samples where more than 40% of the individuals were identified to family level (76-100%: 14 samples, 51-75%: 5 samples, 41-50%: 14 samples). As a result of this processing methodology we readily acknowledge possible underestimations and restrictions within the dataset. Data were presence/absence transformed before the analysis. Hierarchical agglomerative clustering was based on Bray-Curtis similarity formula (Bray and Curtis 1957) using a group average method. SIMPROF test with 1% significance level was performed in order to confirm multivariate structure within the group (Clarke and Gorley 2015). Multivariate statistical analysis was performed using the Primer 7 package. Differences in the number of families per sample between the groups obtained in the Cluster Analysis were tested with use of Mann-Whitney U test in the STATISTICA 6 package.

Additional multivariate analyses (Bray-Curtis formula, group average grouping method, SIMPROF test with 1% significance level) were also carried out for the two families whose specimens were identified to species level (Amphilochidae - 32 samples and Oedicerotidae -25 samples). Here, abundance data (number of individuals per station) were standardised and square root transformed prior to analysis (Clarke and Gorley 2015). Additionally, a set of 39 epibenthic sled (RP sled) samples collected during the BIOICE project was analysed (Amphilochidae to species level, see Suppl. material 2). Similarity analyses were performed following the same statistical methods outlined for the IceAGE Amphilochidae.

## Results and discussion

**Figure 2.**
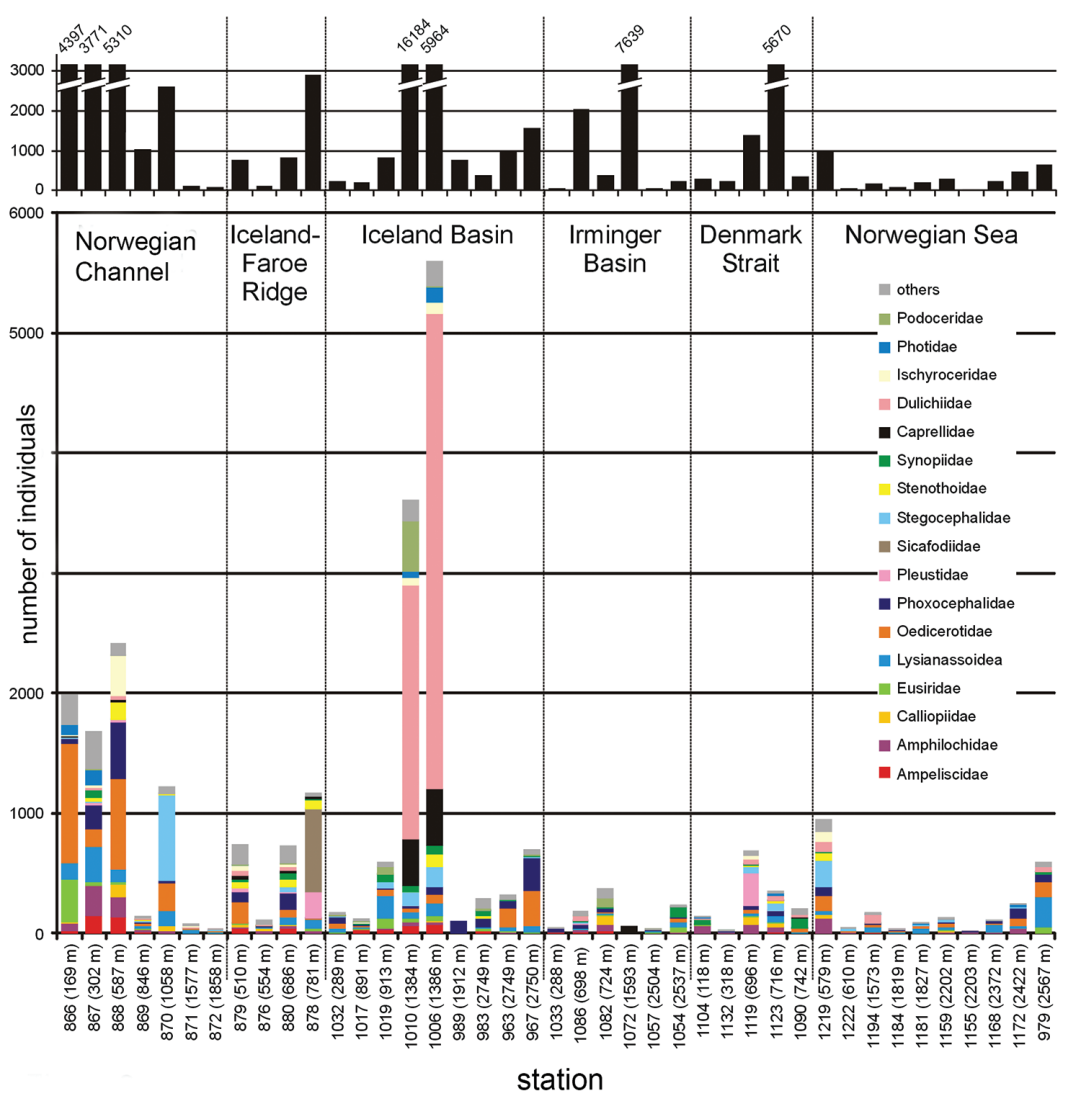
Family distribution at all IceAGE stations ordered by transect. Within each transect (1: Norwegian Channel, 2: Iceland-Faroe Ridge, 3: Iceland Basin, 4: Irmninger Basin, 5: Denmark Strait, 6: Norwegian Sea), stations are ordered by depth. The upper graph (black bars) indicates the absolute number of amphipod individuals per station. The lower graph indicates the amphipods sorted to families per station (legend by colours shown on the right side).

Amphipod crustaceans were collected at all 55 stations analysed; however, identification to the family level was only possible for 40 of them (see Figure 1). The number of individuals per station ranged from a few specimens to more than 16,000 individuals (Figure 2). The Norwegian Sea stations were characterised by low amphipod abundances with higher numbers of individuals found at both the shallowest and the deepest stations. Conversely, in the Norwegian Channel, very high abundances were observed at upper bathyal stations, while the highest numbers of Amphipoda in the Iceland Basin were observed at mid-bathyal stations. In other studied areas, no clear pattern associated with depth was noticed. Amphipoda are known to be an abundant group at all depths. Generally, amphipod abundance is high in the shelf zone and at upper bathyal depths (500-1000 m), while they are generally replaced by the Isopoda at greater depths (Brandt 1997a, Brandt et al. 2005, 2015). In this study, a decrease in amphipod abundance at lower bathyal stations is also observed; however, at shallower stations, the number of individuals seems to depend more on local environmental conditions than on depth.

In numerical order, the most abundant taxa were the Dulichiidae, Oedicerotidae, Phoxocephalidae, and Lysianassoidea followed by the Amphilochidae (Figure 2). All together, these taxa accounted for more than 50% of the individuals studied. It is worth noting that the dominance of dulichiids resulted from their very high abundance at just two mid-bathyal stations in the Iceland Basin (stations 1006, 1010). The overall frequency of occurrence of this family was 60% and, at most other stations, it was represented by a low number of individuals. A large proportion of these same stations also included representatives of the families Caprellidae, Ischyroceridae, Photidae, and Podoceridae. All of these taxa are moderately mobile, and are known to be associated with sessile organisms, often being suspension-feeders (e.g., Lincoln 1979, Caine 1989, Brandt 1993, 1997b). Brix et al. (in press) have reported that at the same stations, high numbers of the isopod family Arcturidae are recorded and these are also regarded as having a sedentary lifestyle, associated with other sessile invertebrates. A study of benthic habitats around Iceland revealed very homogenous sediments in the Iceland Basin, dominated by sandy muds occasionally accompanied by a small proportion of gravel (Mei?ner et al. 2014). However, the region is known to have very productive surface waters and high total organic carbon content in the sediments was observed, which may explain the high abundances of suspension feeding peracarids in our study. The other families that dominated the studied material were more evenly distributed and more frequent (frequency of occurrence often >80%). The numerical dominance of oedicerotids, phoxocephalids, and lysianassoids in the benthic realm is a common feature of both shallow and deep-sea ecosystems in all regions of the World (e.g. Buhl-Jensen 1986, Brandt 1993; Buhl-Mortensen 1996, Weisshappel and Svavarsson 1998, Golovan et al. 2013).

### Acanthonotozomatidae Stebbing, 1906

Figure 3a

In the present study, the family was recorded at two of the 40 stations from ca. 600 m north of Faroe Islands, with a total of ten specimens. In a revision of the Iphimediidae and related families, Coleman and Barnard (1991) limited the family Acanthonotozomatidae to the species of the genus *Acanthonotozoma*. Just (1978) published a taxonomic monograph on this genus including data on biogeography and biology. The World Amphipoda Database (Horton et al. 2017) today lists ten species of Acanthonotozomatidae, of which *Acanthonotozoma cristatum* (Ross, 1835) and *Acanthonotozoma serratum* (Fabricius, 1780) occur around Iceland and *Acanthonotozoma magnum* Just, 1978 and *Acanthonotozoma dunbari* Just, 1978 are known from along the east coast of Greenland and Spitsbergen and South Greenland respectively (Just, 1978). *Acanthonotozoma serratum* seems to be confined to depths less than 200 m, whereas *A. cristatum* has been recorded to 700 m (Just 1978). The colour patterns of species of *Acanthonotozoma* can be vivid, ranging from yellow with red stripes (*A. serratum*) to bright red or even purple (*A. inflatum*). Although Just (1978) provided details on the life history of acanthonotozomatids, further details of the biology, such as feeding preferences are not known.

**Figure 3.**
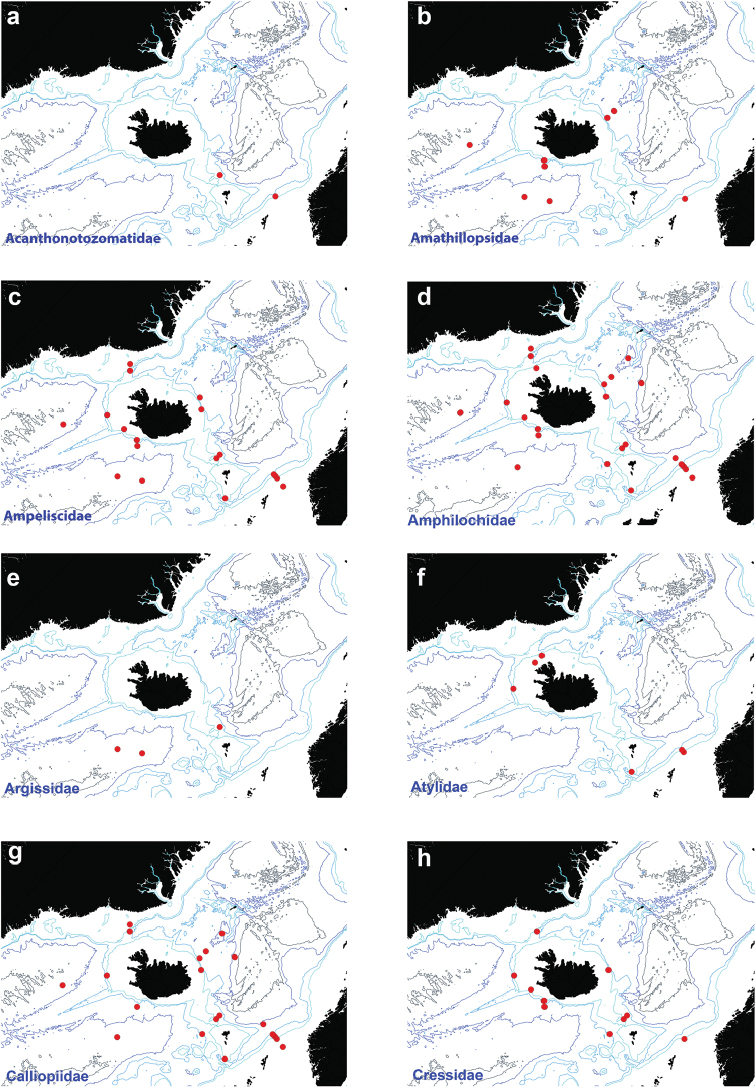
Distribution map for **a** Acanthonotozomatidae **b** Amathillopsidae **c** Ampeliscidae **d** Amphilochidae **e** Argissidae **f** Atylidae **g** Calliopiidae **h** Cressidae in sorted IceAGE EBS samples.

### Amathillopsidae Pirlot, 1934

Figure 3b

In total, 50 amathillopsid individuals were reported from 12 stations, most from the two upper bathyal stations just South of Iceland. *Cleonardopsis* was found at stations west of Iceland whereas *Amathillopsis* is reported from the eastern stations. Forty-six specimens of *Cleonardopsis* were recorded, distributed in the Iceland Basin (eight stations) and the Irminger Basin (one station). The Amathillopsidae consists of a few little known, but morphologically spectacular, large amphipods, which lead a pelagic or bentho-pelagic life. Amathillopsids are found from the Arctic to the Antarctic. In the North Atlantic, the most commonly reported species is *Amathillopsis spinigera* Heller, 1875, with the lesser cited *A. affinis* Miers, 1881 possibly also present. In the present study, four specimens of this genus were recorded at three deep stations in the Norwegian Sea. The genus *Cleonardopsis* was reassigned to the Amathillopsidae in the new subfamily Cleonardopsinae (Lowry 2006). The species *Cleonardopsis carinata* K.H. Barnard, 1916 shows a cosmopolitan distribution in the deep sea. Described from South Atlantic waters (Cape Peninsula area; Barnard 1916), it has also been reported from eastern Greenland (Stephensen 1944), Bay of Biscay (Elizalde et al. 1993, Dauvin and Sorbe 1995, Frutos and Sorbe 2014a) and eastern Indonesia (off the Moluccas; Pirlot 1934).

### Ampeliscidae Kroyer, 1842

Figure 3c

Ampeliscidae were reported from 23 of the 40 stations studied, with a total of 492 specimens. The Ampeliscidae are a benthic, soft sediment, generally tube-dwelling family. This group has strong grain-size and depth constraints (Dauvin et al. 2012). The family is known from intertidal to abyssal depths and often have antenna and pereopod morphology adapted for different feeding strategies at depth (Barnard 1961). The four genera in the family have some delimitation with depth with *Ampelisca*, the most diverse genus with more than 200 species, being a generalist in both depth and habitat requirements. This genus most frequently occurs in shallower waters, and numerous species are recorded from the Atlantic Ocean (Dauvin et al. 2012). A number of papers have recorded and described species from this family from northern Atlantic waters, particularly Scandinavian, Faroe Island and Icelandic waters and consequently there is a wealth of information regarding the depth, sediment, and distributional patterns of the group (Sars 1890-1895, Bellan-Santini and Dauvin 1988, 1997, 2008, Dauvin 1996, Dauvin et al. 2012). Thirteen of the 20 species of Ampeliscidae previously documented from Icelandic waters (Dauvin et al. 2012), were recorded in the IceAGE samples. The majority of ampeliscid species collected are in the genus *Ampelisca*. There were six species from the samples reported as new to Icelandic waters, with three of these new to science (Peart 2018). Relatively low abundances were observed compared to the BIOICE study (Dauvin et al. 2012). The genera *Byblis* (with more than 90 species), *Haploops* (with more than 20 species) and *Byblisoides* (six species) have been more frequently reported from deeper waters (Dauvin et al. 2012). The distribution reported here (Figure 3c) is supported by published data, which reports a wide distribution of Ampeliscidae around Iceland with the majority of the species occurring between 500 and 1500 m.

### Amphilochidae Boeck, 1871

Figure 3d

Amphilochids were reported from 33 of the 40 stations studied, with a total of 1110 specimens. The family Amphilochidae is cosmopolitan and interestingly includes one species (*Gitanopsis alvina* Bellan-Santini and Thurston, 1996) from hydrothermal vents at the Mid-Atlantic Ridge (Bellan-Santini and Thurston 1996). The general body shape of amphilochids is small (1-5 mm mostly) and slightly stout, and observations of living specimens from the Norwegian and Barents seas support the impression that they are not fast swimmers (Tandberg, Vader personal observation). Amphilochids occur at all depths and temperatures, and are quite abundant in the North Atlantic, as in other cold seas. A total of 1110 amphilochid specimens were recorded in the the study, distributed across the genera *Amphilochus*, *Amphilochopsis*, *Amphilochoides*, *Gitana*, *Gitanopsis*, and *Paramphilochoides* (for a discussion on the genus *Amphilochopsis* see Tandberg and Vader (2018)). These specimens constituted 13 of the 17 species of Amphilochidae previously reported from the eastern North-Atlantic and Arctic (Vader and Tandberg, personal communication about a manuscript in preparation). Amphilochidae were found at 33 of the 40 stations that have been processed, with three stations (two in the Norwegian Channel and one north east of Iceland) having more than 100 specimens each. The Amphilochidae were found across all depth and temperature ranges in the IceAGE station network. Previous studies of amphilochids around Iceland indicated that the highest abundance and diversity occurs in the north of Iceland (Weisshappel and Svavarsson 1998).

### Argissidae Walker, 1904

Figure 3e

Argissids were collected at three of the 40 sampling stations, all located south of Iceland, at 686-2749 m, with a total of six specimens. The family Argissidae comprises the single species *Argissa hamatipes* (Norman, 1869), originally described from shallow water in St. Magnus Bay, Shetland Islands, Scotland. Another species, *A. stebbingi* Bonnier, 1896, described from bathyal muddy bottoms of the southern Bay of Biscay, is currently considered a junior synonym of *A. hamatipes*. However, Lowry and Myers (2017) note that the genus *Argissa* is in need of revision since the distribution, depth range and morphological variation attributed to *A. hamatipes* are implausible when attributed to a single species. In the southern Bay of Biscay, *A. hamatipes* was collected with a suprabenthic sledge on sandy and muddy sand bottoms of the continental shelf (31-179 m), with a decreasing frequency of occurrence with depth (Sorbe 1984) and also at bathyal depths (711-1098 m) on muddy bottoms (Dauvin and Sorbe 1995, Frutos and Sorbe 2014a, Sorbe and Elizalde 2014). *Argissa hamatipes* is known to occur from the northeastern Atlantic Ocean and Norwegian Arctic (Palerud and Vader 1991). According to Wildish and Peer (1981) *A. hamatipes* is a deposit feeder. Given the current monotypic status of the family, this material is assigned to *A. hamatipes* extending the bathymetric distribution of this species to 2749 m, and supporting the need for a revision of the genus *Argissa*.

### Atylidae Lilljeborg, 1865

Figure 3f

Atylidae occurred in samples from the Denmark Strait and the Faroe Channel at stations associated with strong bottom currents, in six of the 40 stations studied, with a total of 20 specimens. Palerud and Vader (1991) reported seven species of North-Atlantic Atylidae, currently ascribed to the genera *Atylus* and *Nototropis* (see Bousfield and Kendall 1994). Atylids are sometimes larger than 10 mm (Hendrycks, personal communication) and show particularly strong lateral compression along the dorsal pereonites with some species with middorsal carinae. Characteristic for this taxon is a notch in the dorsal keel of urosomite 1. The cuticle of atylids in most cases is thin and yellowish or unpigmented. Little is known of the ecology of atylids, though they appear to occur more commonly in soft bottom shallow-water habitats.

### Calliopiidae Sars, 1893

Figure 3g

The family was present at 24 of the 40 stations studied, with a total of 470 specimens indicating its relative importance in this cold-water area. The relatively speciose family Calliopiidae is represented in the north east Atlantic by 39 species from 12 genera (Vader and Tandberg, unpublished data) which accounts for almost 50% of the known Calliopiidae in the world (Horton et al. 2017). Calliopiidae appear to favour colder waters, although many species are known from more temperate waters, few (if any) are found in warm waters (Bousfield and Hendrycks 1997). Several calliopiid species are known to be associates of molluscs, crustaceans (Vader and Tandberg 2013, 2015) and sponges (Vader 1984, Amsler et al. 2009). Non-associate calliopid species are found on sandy or muddy seafloors and in macroalgae from littoral to bathyal depths (d'Udekem d'Acoz 2012). One species, *Apherusa glacialis* (Hansen, 1888) is associated exclusively with sea-ice habitat. Most species are carnivorous or detritivorous (Bousfield and Hendrycks 1997). Weisshappel (2001) showed a distinct difference in the species composition of the Calliopiidae on different sides of the Greenland-Iceland-Faroe ridge, with 72% of the species being restricted to one side of the ridge.

### Cressidae Stebbing, 1899

Figure 3h

The family Cressidae was reported from 14 of the 40 stations studied, with a total of 190 specimens. Cressids have a compact body, specialised mandible and an extremely lengthened mandibular palp (Krapp-Schickel 2005). The family Cressidae is found mainly in the northern regions of the Atlantic and is comprised of ten species in two genera, *Cressa* Boeck, 1871 and *Cressina* Stephensen, 1931 (Horton et al. 2017). Three species have been identified so far as part of the IceAGE program, the same species as were indicated by Stephensen (1931) namely: *Cressa carinata* Stephensen, 1931, *Cressa minuta* Boeck, 1871 and *Cressina monocuspis* Stephensen, 1931.

### Cyproideidae Barnard, 1974

Figure 4a

Cyproideidae was reported from three of the 40 stations studied. Twenty-four cyproideid specimens were recorded in the IceAGE material, mainly on the upper slope in both Irminger and Iceland basins (13 and ten individuals, respectively). A single specimen was also recorded from a northern Faroe station (station 879). The Cyproideidae are characterised by immensely broadened coxae 3/4 with contiguous abutting margins and overlapping coxae 1/2. The Cyproideidae includes 20 genera with 46 species (Horton et al. 2017). Cyproideids are found in association with marine algae, intertidal rocks or coral debris (Barnard 1972b, Lowry and Stoddart 2003, Azman 2009). They are also known to have associations with live corals (Myers 1985, Thomas 1999), sponges (Ortiz et al. 2000) and crinoids (Lowry and Azman 2008). Cyproideids are most diverse in the littoral shallow marine waters of the Indo-West Pacific (Barnard and Karaman 1991, Lowry and Azman 2008, Ariyama 2016), with just two genera recorded in the north east Atlantic: *Peltocoxa* and *Stegoplax*. *Peltocoxa* comprises five species, two of which (*P. brevirostris* (Scott and Scott, 1893) and *P. damnoniensis* (Stebbing, 1885)) occur in the Atlantic (Lincoln 1979, Palerud and Vader 1991). *Stegoplax* comprises a single deep-sea species, *Stegoplax longirostris* Sars, 1882 with a boreal distribution (Sars 1883, 1890-1895, Stephensen 1925, 1938, Buhl-Jensen 1986, Palerud and Vader 1991).

**Figure 4.**
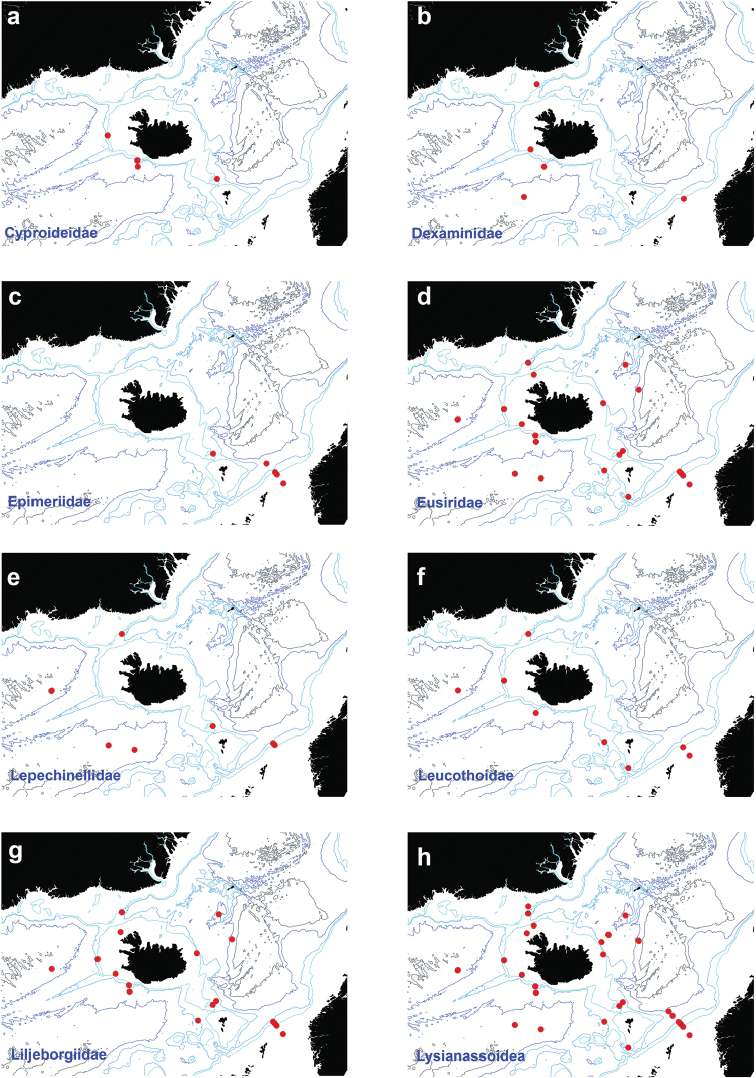
Distribution map for **a** Cyproideidae **b** Dexaminidae **c** Epimeriidae **d** Eusiridae **e** Lepechinellidae **f** Leucothoidae **g** Liljeborgiidae **h** Lysianassoidea, in sorted IceAGE EBS samples.

### Dexaminidae Leach, 1814

Figure 4b

Dexaminids were found at four of the 40 stations studied in shallow areas east of Faroe Islands, Iceland Basin and Denmark Strait and at one deep station (2749 m) in the Iceland Basin, with a total of 14 specimens. Four species of the family Dexaminidae have been reported in the North-Atlantic (Palerud and Vader 1991). Dexaminids usually have carinate pleon segments, except for the genus *Guernea*, and their urosome segments 2 and 3 are fused. *Dexamine spinosa* (Montagu, 1813) and *Dexamine thea* Boeck, 1861 occur in shallow-water to 60 m (Lincoln 1979). *Tritaeta gibbosa* (Spence Bate, 1862) is associated with various invertebrates, living in sponges and ascidians (Lincoln 1979), and has also been shown to live in pouches in the integument of holothurians (Laetz et al. 2013). The Greenlandic *Guernea nordenskioldi* (Hansen, 1888), recently found both in Russia and in Svalbard waters, may well occur in the IceAGE samples. The short bodied *Guernea coalita* (Norman, 1868), a more southern species, is fossorial and occurs in fine sediments (Kim et al. 2011).

### Epimeriidae Boeck, 1871

Figure 4c

Epimeriidae were reported from five of the 40 stations studied, with a total of 55 specimens, in the area of Iceland-Faroe Ridge, at depths less than 1600 m. This family usually feature prominent teeth carinae (d'Udekem d'Acoz 2010, Krapp-Schickel 2011) and/or robustly elongated coxal plates (Moore 1981, Barnard and Karaman 1991). Members of the Epimeriidae are bottom-dwelling, epibenthic amphipods represented by two genera in the northern Atlantic, *Epimeria* and *Paramphithoe* (Palerud and Vader 1991, Horton et al. 2017). Although the species are not abundant, this readily recognisable family are frequently recorded from shallow to deep waters of northern Atlantic and Arctic waters as well as around Iceland. Epimeriids contain members of several feeding types, such as filter feeders and micro-predators (Dauby et al 2001).

### Eusiridae Stebbing, 1888

Figure 4d

Eusiridae were reported from 27 of the 40 stations studied with representatives in all sampling areas and depth zones. A total of 775 specimens of Eusiridae have so far been identified from the IceAGE samples. Eusirids are abundant members of the deep-sea fauna off Iceland (Weisshappel 2000), known to be predators (Enequist 1949) with good swimming capabilities (Bousfield and Hendrycks 1995). *Rhachotropis* Smith, 1883 was the dominant genus (see Lorz et al. 2018), with three other genera represented; namely *Eusirus, Cleonardo*, and *Eusirella*. About half of the specimens collected, 355 individuals, were from a single station at 169 m, which was also the shallowest sampled (station 866). This easternmost station is at the edge of the North Sea in contrast to other stations containing eusirids, which are in the Arctic waters of the Norwegian Greenland Seas. This shallow station was dominated by a small *Rhachotropis* species with large eyes, *Rhachotropis northriana* d'Udekem d'Acoz, Vader & Legezynska, 2007.

### Lepechinellidae Schellenberg, 1926

Figure 4e

Lepechinellids were reported from ten of the 40 stations studied, with a total of 103 specimens. The lepechinellids are well adapted to a demersal or epibenthic lifestyle on soft substrates in deeper waters (Barnard 1973). The family Lepechinellidae comprises five genera, three of which (*Lepechinella*; *Lepechinelloides*; *Lepesubchela*) have been reported in our study area (Thurston 1980, Palerud and Vader 1991, Johansen and Vader 2015). The genus *Lepechinella* was the most speciose and abundant taxon in the IceAGE samples. More than half of the lepechinellids collected, 60 individuals, were sampled from a single station at 500 m depth at the Iceland-Faroe Ridge (station 879). Owing to the fragility of the slender spines and thin elongate pereopods characteristic of the taxon, intact lepechinellids are difficult to obtain; however, the majority of specimens collected were considered to be in good condition. Morphological characters are known to vary strongly with growth and gender amongst lepechinellids (Barnard 1973, Thurston 1980). The present samples obtained a range of sizes of both genders of *Lepechinella arctica* Schellenberg, 1926, providing a promising opportunity for further studies on this species.

### Leucothoidae Dana, 1852

Figure 4f

Leucothoids were reported from nine of the 40 stations studied, with a total of 35 specimens. The Leucothoidae are well represented within the Atlantic Ocean, though mainly in warmer regions. The documented Atlantic Ocean leucothoids have broad shared distributions with eight species also known from South and West Africa, 13 from the Caribbean and Gulf of Mexico, five from Brazil, three species from the Azores, Biscay, and Mid-Atlantic Ridge near Santa Cruz das Flores, and seven species from United Kingdom waters to the central and northern Atlantic. A new species of leucothoid is described based on specimens collected during the IceAGE expedition (Krapp-Schickel 2018). Leucothoids are usually found near, with, or in sponges or tunicates, and thus specimens are often overlooked inquilines (White 2011). During processing of IceAGE material, only juvenile and male individuals attributable to three species were identified, namely to the *Leucothoe spinicarpa* complex, *L. lilljeborgi* Boeck, 1861 and *L. vaderotti* Krapp-Schickel, 2018 (Krapp-Schickel 2018).

### Liljeborgiidae Stebbing, 1899

Figure 4g

Three hundred eleven liljeborgiid specimens were collected from 20 of the 40 stations studied and covering a range of depths and distinct hydrological features. The Liljeborgiidae are micropredators, with some being known associates of other invertebrates, including hermit crabs (Vader 1995). Thirteen species of Liljeborgiidae have been reported from the North Atlantic (Vader and Tandberg, unpublished data). Liljeborgiids are primarily benthic species and can occasionally be quite abundant in benthic samples. A single station at 303 m depth in the Norwegian Trench (station 867) was characterised by an extremely high abundance of Liljeborgiidae (113 individuals).

### Lysianassoidea Dana, 1849

Figure 4h

A total of 2008 specimens of lysianassoids and allied taxa was reported from 38 of the 40 stations studied occurring at depths from 169 to 2743 m. This superfamily is an incredibly large, diverse group of amphipods, which includes scavengers, predators, ectoparasites, obligate associates, and inquilines (e.g., Lowry and Stoddart 1983). The recent revision of Lowry and Myers (2017) has greatly restricted the concept of the Lysianassoidea to 130 genera in 12 families, where formerly the superfamily was composed of 22 families, 173 genera, and 1042 species. Lysianassoids range from a few millimetres in body length to the largest known amphipod, the 34 cm plus *Alicella gigantea* (Chevreux, 1899) which occurs in the deep North Atlantic and Pacific. Lysianassoidea are distributed globally and are particularly abundant at depth, where they form a specialist necrophagous guild, feeding on large and small food-falls (Horton and Thurston 2013). Many lysianassoids are highly mobile, fast swimmers, detecting food-falls from long range through chemoreception (e.g., Premke et al. 2003). Vader and Tandberg (unpublished) list almost 200 species of lysianassoid and allied taxa (including the Alicellidae, Scopelocheiridae, Valettiopsidae, and Eurytheneidae) from the eastern North-Atlantic and Arctic. The great diversity and abundance of lysianassoid taxa identified in the IceAGE material precluded anything more than a cursorial observation during the workshops and a sample set of this size is certainly worthy of a more in-depth study.

### Melphidippidae Stebbing, 1899

Figure 5a

In total, 254 melphidippid specimens were recorded from 16 of the 40 stations. Melphidippids have elaborate spination and elongate slender legs and are, at least partially, epifaunal. Several studies have indicated that the normal orientation is upside down in a sling created by the elongate pereopods V to VII (Enequist 1949). Four species of the family Melphidippidae are known from the Nordic Seas, *Melphidippa borealis* Boeck, 1871, *M. goesi* Stebbing, 1899, *M. macrura* Sars, 1894 and *Melphidippella macra* Norman, 1869. The most common species is *Melphidippa borealis*, which has a wide depth distribution, from 50 to 2300 m. *Melphidippa goesi* is a more northerly species and is rarer on the Norwegian shelf, yet is frequently found off Iceland at 68 to 688 m. The closely aligned *Melphidipella macra* and *Melphidippa macrura* have more southerly distributions. *Melphidipella macra* has not been recorded from Iceland, is rarely recorded from Norwegian waters but is common in the Skagerrak (Miskov-Nodland et al. 1999). *Melphidippa macrura* is known only from Icelandic waters where water temperatures exceed 3 °C. Distribution of individuals appeared to indicate aggregations with numerous specimens at some stations, up to 83 individuals. Similar high-density records are also reported for *M. willemiana* d'Udekem d'Acoz, 2006, off Svalbard.

**Figure 5.**
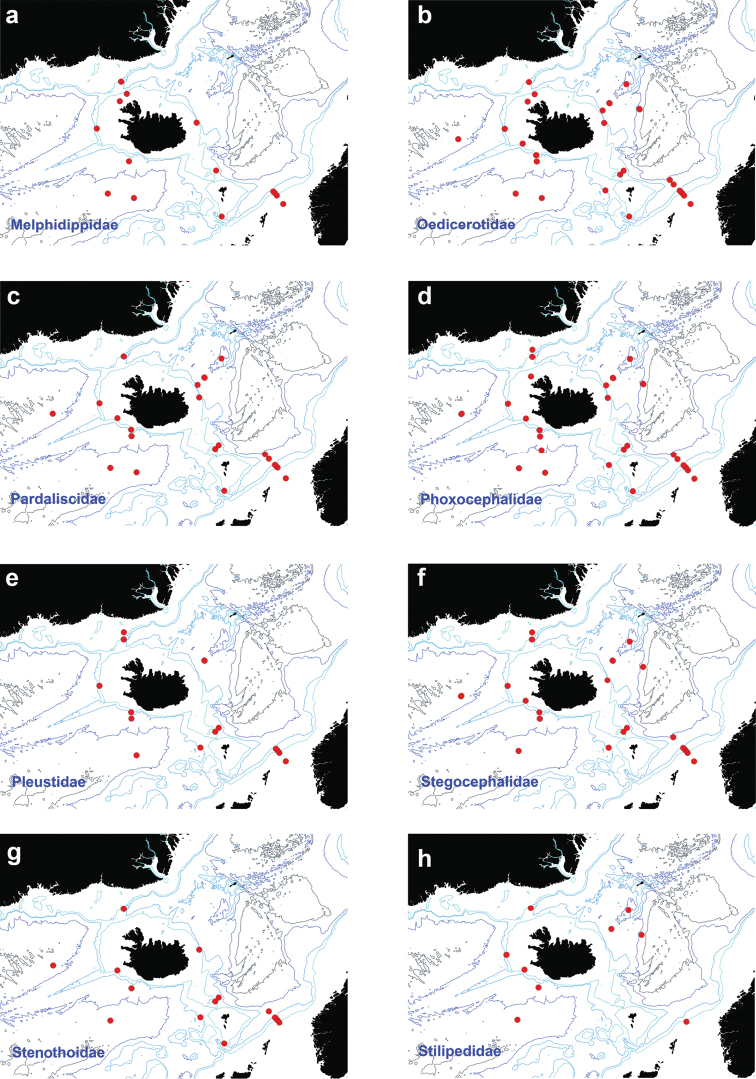
Distribution map for **a** Melphidippidae **b** Oedicerotidae **c** Pardaliscidae **d** Phoxocephalidae **e** Pleustidae **f** Stegocephalidae **g** Stenothoidae **h** Stilipedidae, in sorted IceAGE EBS samples.

### Oedicerotidae Lilljeborg, 1865

Figure 5b

Oedicerotids were present and often the most abundant family at 35 of the 40 stations and occurred alongside other fossorial amphipod families including the Phoxocephalidae and Urothoidae. A total of 3569 specimens was reported; nine genera and 21 species were identified from the material. Among amphipods, the Oedicerotidae are a dominant part of the North Atlantic benthic fauna. Oedicerotids live within the surface sediment of the seafloor and are deposit feeders (Enequist 1949) or can be carnivorous (Oliver and Slattery 1985). Shallow-water species are known to migrate into the water column for reproduction around lunar cycles and with tidal rhythms (Alldredge and King 1977, Forward 1986).

### Pardaliscidae Boeck, 1871

Figure 5c

Pardaliscids occurred at 26 of the 40 stations studied, with a total of 327 specimens, suggesting that the family is well represented in the North Atlantic. Pardaliscids contain approximately 80 species worldwide (Horton et al. 2017). The family Pardaliscidae have good swimming ability and are mostly found living in deep-sea habitats (Birstein and Vinogradov 1962, Karaman 1974). A few genera are considered benthic, e.g., *Epereopus*; however the majority are thought to range between demersal and epibenthic with the ability to move far up in the water column. Assessment of gnathopod and mouthpart morphology implies that pardaliscids are a predatory family (Hendrycks and Conlan 2003). Weisshappel and Svavarsson (1998) reported pardaliscids from both north and south of Iceland, but the diversity was greater in the southern sites.

### Phoxocephalidae Sars, 1891

Figure 5d

Phoxocephalidae were reported from 39 of the 40 stations studied, with a total of 2134 specimens. The family Phoxocephalidae contains 375 species (Horton et al. 2017) recorded in all oceans from tropical to polar zones with very high diversity in Australian waters (Barnard and Karaman 1991). Phoxocephalids are fossorial and burrow within soft sediments (De Broyer et al. 2003) constituting an abundant part of the infaunal amphipod assemblages from the shallow sublittoral to the deep sea (Cummings et al. 1998, Lorz and Bamber 2010, Jazdzewska 2015). They are predators (Oliver et al. 1982, Oliver and Slattery 1985). In the North Atlantic, Palerud and Vader (1991) reported 21 phoxocephalid species ascribed to seven genera. Phoxocephalids are particularly abundant around Iceland (Brandt 1993, Brandt and Piepenburg 1994, Weisshappel and Svavarsson 1998) and the Norwegian Sea (Buhl-Jensen 1986, Buhl-Mortensen 1996).

### Pleustidae Buchholz, 1874

Figure 5e

Pleustidae were reported from 16 of the 40 stations studied, with a total of 594 specimens. The family Pleustidae currently contains 241 species worldwide (Horton et al. 2017). Pleustid species are mostly small to medium-sized (range from 4-20 mm, but most are approx. 10 mm or less), benthic detritivores and carnivores (Bousfield and Hendrycks 1994). Many of the pleustid subfamilies have members which are closely associated with other invertebrates. Globally the distribution of pleustids is mainly Holarctic, North Atlantic and Arctic with only a small group recorded in southern hemisphere waters. The diversity of pleustids is most likely related to the abundance of other benthic invertebrates at the sites, as well as availability of algae for substrate. This potential inquiline association is supported by a patchy distribution, where two of the 15 IceAGE stations contained more than 200 individuals.

### Stegocephalidae Dana, 1852

Figure 5f

In total, 1552 stegocephalids were found at 29 of 40 stations studied. Four of these stations reported more than 100 specimens, including one with 704 individuals (station 870). Stegocephalidae have been found at all depths and temperature ranges in the IceAGE material which aligns with the findings of the BIOICE expedition (Berge and Vader 1997). The family Stegocephalidae is common in the North Atlantic and contains 26 genera and more than 100 species (Horton et al. 2017). Stegocephalids are quite variable in size, with the largest species found in the coldest waters. Most species are benthopelagic, while a few species (*Parandania* spp.) are truly pelagic. These latter are caught only irregularly, while some of the benthopelagic species such as *Andaniexis* and *Andaniopsis* may occur in large numbers over deep soft bottoms (Sars 1895, Vader pers. comm). Stegocephalids feed mainly as micro-predators on large invertebrates, quite often coelenterates, but some species are also predators, and a few live in loose associations with other invertebrates (Vader 1984). In the lower latitudes of the North Atlantic, 24 species have been reported (Vader, unpublished data).

### Stenothoidae Boeck, 1871

Figure 5g

A total of 500 stenothoid specimens were recorded from 39 of the 40 stations. Stenothoids are well known associates of molluscs, sponges, or coelenterates (Krapp-Schickel and Vader 2015). While some probably profit from the water current created by the host, enabling them to filter-feed or graze their epiphytes, others are known to feed directly on tissues of the host's body or entire polyps. Thus, some species are found only by examination of the host, and not by sledge or trawl sampling methods. The diversity of Stenothoidae is high in the Atlantic Ocean with many species of *Stenothoe* (more species in shallower waters), *Metopa* (more species in deeper regions), and some *Stenula* species found in the study region. Thirty-five species of *Metopa* and ~ 20 species of *Stenothoe* occur in the Atlantic Ocean and Arctic. Two genera, *Metopa* and *Stenothoe* were found abundantly within the IceAGE collections. A third genus, *Stenula*, is rarely present.

### Stilipedidae Holmes, 1908

Figure 5h

A total of 30 stilipedids was sampled from ten of the 40 stations studied. The family Stilipedidae is divided into three subfamilies and comprises six genera (*Alexandrella*, *Astyra*, *Astyroides*, *Bathypanoploea*, *Eclysis* and *Stilipes*) (Horton et al. 2017). The Stilipedidae is a cosmopolitan family, and only the genera *Astyra* and *Stilipes* occur in the NE Atlantic. An undescribed bathyal *Stilipes* species has been recorded in temperate waters of the Bay of Biscay (Lagardere 1977, Sorbe and Weber 1995, Frutos and Sorbe 2014a); whereas two *Astyra* species (*A. abyssi* Boeck, 1871 and *A. longipes* Stephensen, 1933) have been reported in boreal waters of Greenland-Iceland-Faroe and Norwegian seas (Stephensen 1931, 1933, 1940, Palerud and Vader 1991, Brandt 1993, Brandt and Piepenburg 1994, Brandt et al. 1996). The genus *Stilipes* and some *Astyra* species are apparently pelagic (Berge 2003). The sampling carried out with sledges in the near-bottom environment (Sorbe and Weber 1995, Frutos and Sorbe 2014b, present study) shows these species could also exhibit a suprabenthic behaviour. *Astyra abyssi* was the most frequently found species (24 specimens), sampled at five stations in the Irminger and Iceland basins and in the Norwegian Channel; whereas *A. longipes* occurred in deeper water in the Norwegian Sea. A different (possibly new) *Astyra* species, was recorded in the Iceland basin at the deepest station (>2700 m) of the expedition (station 967).

### Synopiidae Dana, 1853

Figure 6a

Synopiidae were sampled at 19 of the 40 stations studied, with a total of 676 specimens. The Synopiidae are a typical deep-sea family distributed worldwide (Barnard 1972a). Synopiids can be easily recognised by the large head and rostrum shape, feebly developed gnathopods, and very large telson. Currently, the Synopiidae comprises 108 species in 18 genera (Horton et al. 2017). Synopiids in NE Atlantic waters are represented by the genera: *Austrosyrrhoe*, *Bruzelia*, *Ileraustroe*, *Jeddo*, *Pseudotiron*, *Stephobruzelia*, *Syrrhoe*, *Syrrhoites*, and *Tiron* (Sars 1890-1895, Stephensen 1931, 1938, 1944, Buhl-Jensen 1986, Palerud and Vader 1991, Brandt et al. 1996, Brandt 1997b, Bachelet et al. 2003, Frutos and Sorbe 2014a). They occur in all the areas studied from the Icelandic shelf and slope to the deeper Norwegian, Irminger and Icelandic basins, the Denmark Strait, and the Faroe Channel. At least 13 species belonging to the genera *Austrosyrrhoe*, *Bruzelia*, *Syrrhoe*, *Syrrhoites*, and *Pseudotiron* have been identified so far. Preliminary identifications show that *Syrrhoites* appears to be the most speciose genus.

**Figure 6.**
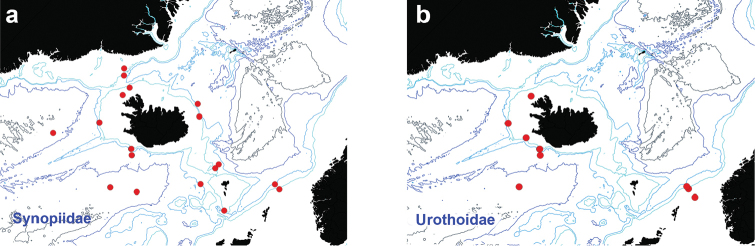
Distribution map for **a** Synopiidae **b** Urothoidae, in sorted IceAGE EBS samples.

### Urothoidae Bousfield, 1978

Figure 6b

A total of 138 individuals were present at 12 of the 40 stations examined, all situated south of Iceland (but were not found in the Norwegian Sea). This family comprises amphipods with small body size, 2 mm to 10 mm, which are highly adapted to a fossorial lifestyle (Bousfield 1982). Urothoidae currently comprises 61 species in six genera (Horton et al. 2017). The family has a cosmopolitan distribution and can be found from shallow waters to abyssal depths (Sittrop et al. 2015). Almost all North Atlantic urothoids are shallow water, sandy bottom species; only *Urothoe elegans* (Spence Bate, 1857) and *Carangolia barnardi* Jaume & Sorbe, 2001 occur in deep North Atlantic waters (Lincoln 1979, Jaume and Sorbe 2001). Urothoids are detritus feeders, and their association with soft bottom habitat supports this. The family is commonly encountered in North Atlantic samples where this habitat dominates.

### Corophiida

**Figure 7.**
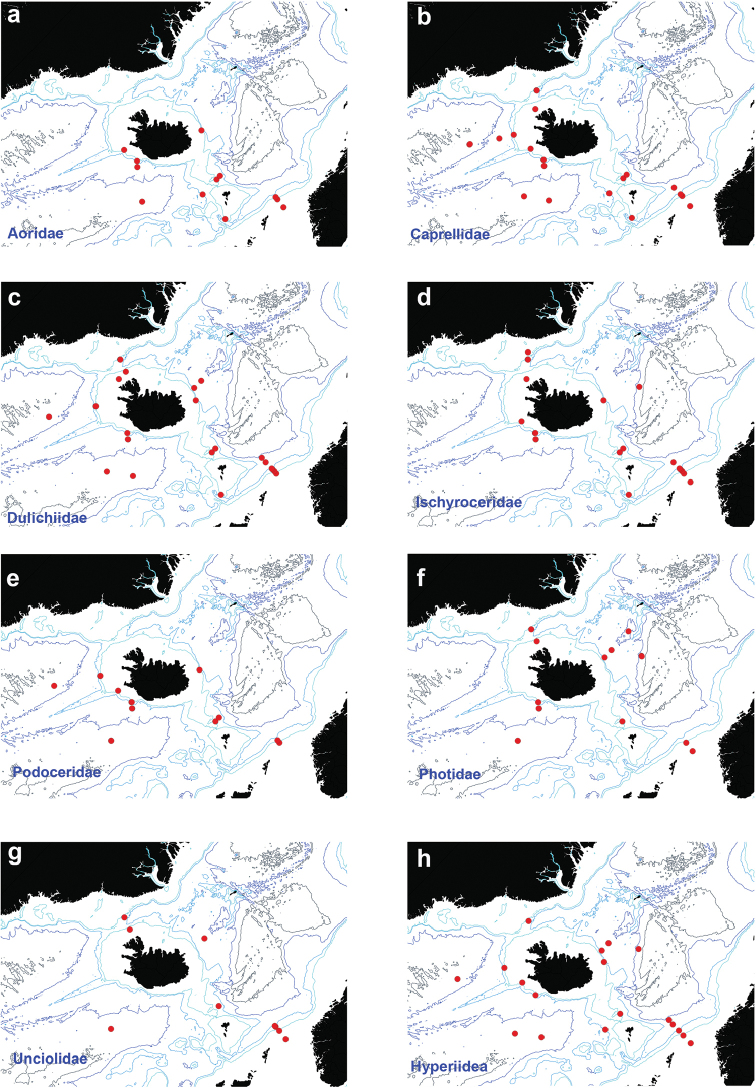
Distribution map for Corophiida **a** Aoridae **b** Caprellidae **c** Dulichiidae **d** Ischyroceridae **e** Podoceridae **f** Photidae **g** Unciolidae **h** Hyperiidea, in sorted IceAGE EBS samples.

Taking into account frequent damage of fragile Amphipoda belonging to the infraorder Corophiida, individuals with uncertain family assignation, are presented in Figure 7.

### Aoridae Stebbing, 1899

Figure 7a

Aorids were collected at 12 of the 40 stations, located east, south and west of Iceland (apparently absent at the northern stations), at depths between 168 and 2750 m, with a total of 105 specimens. The family Aoridae contains 250 known species world-wide belonging to 25 genera. According to Palerud and Vader (1991) and Myers (1998), only 15 aorid species belonging to six genera were listed from the North Atlantic and Norwegian Arctic (including Icelandic waters). *Aora*, *Lembos*, and *Microdeutopus* are detritus-feeders and show tube-dwelling habits, whose construction involves secretions produced by glands located on the third and fourth pairs of pereopods (Enequist 1949).

### Caprellidae Leach, 1814

Figure 7b

Caprellidae occurred at half of the stations sampled (18 of 40 stations), between 168 and 2747 m, with a total of 1052 specimens. The family Caprellidae is large with 91 genera and more than 400 species. Species are often epibionts, associated with other organisms such as algae, hydrozoans, bryozoans (Caine 1989), or even commensals of some marine invertebrates including echinoderms (Guerra-Garcia 2001, Guerra-Garcia et al. 2008) and decapods (Martin and Pettit 1998). As in most groups, knowledge of the biology and distribution is more extensive for shallow-water species; but they are also known to have a significant presence in deep-sea ecosystems, with numerous records from Pacific, Atlantic, Arctic, and circum-Antarctic waters (see Guerra-Garcia 2003). In recent years, the number of species reported from the deep-sea has increased, through the revision of collections from different museums (Guerra-Garcia 2003, 2004, Guerra-Garcia and Garcia-Gomez 2003) and new oceanographic expeditions (Laubitz and Sorbe 1996, Guerra-Garcia et al. 2008).

### Dulichiidae Dana, 1849

Figure 7c

Dulichiids were moderately abundant at more than half the stations in all areas studied and were found in very large numbers (thousands of individuals) at two stations in the Iceland Basin. They were reported from 24 of the 40 stations studied, with a total of 6547 specimens. In our material, Dulichiidae are represented by the genus *Dulichiopsis*, and mainly by the species *Dulichiopsis macera* (Sars, 1879). It is one of the most abundant groups, dominating at stations in the Iceland Basin. The Dulichiidae currently comprises six genera (Rauschert 1990, Horton et al. 2017) and a total of 26 known species. The genus *Dulichiopsis*, is one of the most speciose, with seven described species that are known mainly from the deep sea (183 to 3229 m) being widely distributed in the North Atlantic, North Pacific and Arctic Oceans as well as in the Indian Ocean (north Madagascar) (Laubitz 1977, 1979, Ledoyer 1986). The presence of glandular pereopods 3-4 and very long, slender pereopods 5- suggests that they are filter-feeders and stem-builders. Such behaviour, including self-constructed stems has been described in various coastal species including *Dulichia falcata* (Spence Bate, 1857), *D. rhabdoplastis* McCloskey, 1970, *Dyopedos monacanthus* (Metzger, 1875), *D. porrectus* Spence Bate, 1857 (see McCloskey 1970, Laubitz 1977, 1979, Moore and Earll 1985, Mattson and Cedhagen 1989, Meyer-Rochow et al. 1991, Thiel 1997, 1998), and *Dulichiopsis dianae* Corbari & Sorbe, 2017.

### Ischyroceridae Stebbing, 1899

Figure 7d

Ischyrocerids were reported from 20 of the 40 stations studied, with a total of 731 specimens. The family Ischyroceridae is a diverse group with 269 described species worldwide (Horton et al. 2017). In the north east Atlantic the family is represented by 28 species (Palerud and Vader 1991, Brandt 1997b). Ischyroceridae are mostly suspension- and deposit-feeders and tube-dwellers occurring mainly on the shelf, although some species occur also at bathyal and abyssal depths (Lincoln 1979, Brandt 1993, 1997b). Their key feature is the ability to construct tubes with 'amphipod silk'. Accordingly, hemi-sessile species may occur on soft- and hard-substrata of the northern Atlantic and the Arctic where they can be locally quite abundant (Sars 1893, Barnard and Karaman 1991, Buhl-Mortensen 1996). In the present study, the family was recorded from all areas except the Irminger Basin. Findings from the deeper waters around Iceland, however, were infrequent and restricted to few species (Raitt 1938, Stephensen 1940).

### Podoceridae Leach, 1814

Figure 7e

In this study, 638 podocerids were identified from 11 of the 40 stations studied, with clusters in the waters south-west and south-east of Iceland. This diversity is almost certainly underrepresented, as some specimens were most likely identified as corophiids. The Podoceridae have undergone major changes due to the work of Myers and Lowry (2003). The family presently includes eight accepted genera with ~ 100 species and subspecies, the vast majority belonging to the genus *Podocerus* (Horton et al. 2017). Most members of the Podoceridae inhabit temperate and warm waters and are bottom-living genera with depressed and cylindrical bodies; however, both *Xenodice* and *Neoxenodice* are primarily cold-water amphipods. Podocerids are often found as epifauna on macroalgae and large invertebrates such as sponges and ascidians. They are poor swimmers, with the main method of locomotion being crawling and climbing, with the abdomen flexed under the body (Laubitz 1979).

### Photidae Boeck, 1871

Figure 7f

Photids were collected at 12 of the 40 stations studied located all around Iceland, excluding the Irminger Basin, at depths between 118 and 2749 m, with a total of 454 specimens. Worldwide, the Photidae contain 163 known species belonging to 17 genera (Horton et al. 2017). According to Stephensen (1933, 1940) and Palerud and Vader (1991), 13 photid species belonging to four genera were listed from the north eastern Atlantic and Norwegian Arctic (including Icelandic waters). With few exceptions, Photidae are known to live in littoral and sublittoral habitats reaching shallow to bathyal depths. In the North Atlantic, *Photis longicaudata* (Spence Bate, 1862) and *Photis reinhardi* Kroyer, 1842 construct short tubes of clay or detritus attached to a firm substratum forming dense aggregations along the seafloor (Enequist 1949).

### Unciolidae Myers & Lowry, 2003

Figure 7g

A total of 155 specimens of the family Unciolidae was recovered from ten of the 40 stations studied, in all areas and with a very wide depth range (118-2749 m). Unciolidae are comprised of 18 genera and are distributed worldwide in both cold and warm waters. There are two genera in the subfamily Unciolinae present in Nordic Seas, *Neohela* and *Unciola*. One of the largest and most conspicuous species is *Neohela monstrosa* (Boeck, 1861). It is common in the cold and deep waters of the Norwegian Sea from 300 to 2000 m, and is known to create burrows 10 cm deep and form dense populations on soft deep-sea sediments (Buhl-Mortensen et al. 2016). Another common unciolid, *Unciola planipes* Norman, 1867, is recorded from Skagerrak to north of Lofoten (Vader et al. 1997) and is found below 400 m on the outer parts of the Norwegian shelf (Buhl-Jensen 1986). Other *Unciola* species found in Nordic Seas include *U. crenatipalma* (Spence Bate, 1862) a southerly species not common in Norwegian waters, *U. leucopis* (Kroyer, 1845) and *U. petalocera* (Sars, 1876), which have a northern distribution, found rarely in the Barents Sea (Vader et al. 1997).

### Hyperiidea Milne Edwards, 1830

Figure 7h

Hyperiids were reported from 22 of the 40 stations studied, with a total of 134 specimens. The Hyperiidea is a diverse planktonic suborder of amphipods comprising almost 300 species in 76 genera (Horton et al. 2017). During the IceAGE sampling, specimens of the Hyperiidea were mostly found in low numbers (1-5 individuals per station). Specimens occurred in remarkably high numbers at two stations situated west of the Norwegian shelf break, in the Norwegian Channel, at around 1000 m. Hyperiids are often parasitic or commensal on gelatinous zooplankton (Laval 1980). The IceAGE sampling was carried out by means of an epibenthic sledge and the presence of high numbers of hyperiids caught above the seafloor, indicates their hyperbenthic feeding habits. These habits may be frequent and Vinogradov (1999b) has reported swarms of Arctic *Themisto* feeding on particles on the deep-sea floor. Hyperiids seem to be commonly occurring throughout the Norwegian Channel and were found in high numbers at around 2700 m depth in the Iceland Basin. All specimens were recovered from depths greater than 600 m.

## Statistical Analysis

Within the benthic deep-sea invertebrate assemblages, amphipods are an abundant and diverse group. Worldwide around 10,000 species are described, about 80% of which are marine (Horton et al. 2017). As the most abundant and diverse crustacean order in the marine benthos, the determination of IceAGE amphipod specimens provided urgently needed baseline data to understand the scale of existing collections and for future studies in the North Atlantic and Nordic seas.

### Family level data

**Figure 8.**
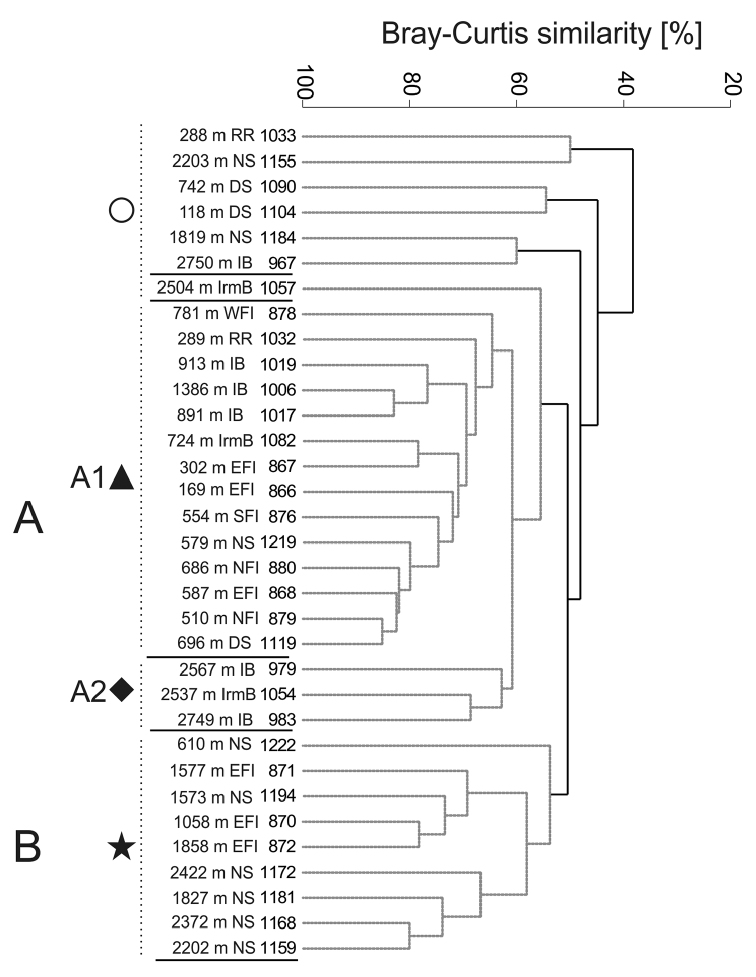
Dendrogram of samples for the family level data (Bray-Curtis similarity, group average grouping method and presence/absence transformed data). Abbreviations: RR - Reykjanes Ridge, NS - Norwegian Sea, DS - Denmark Strait, IB - Iceland Basin, IrmB - Irminger Basin, WFI - west off Faroe Islands, EFI - east off Faroe Islands, SFI - South of Faroe Islands, NFI - North of Faroe Islands. (Grey spotted lines indicate the samples that cannot be significantly differentiated by SIMPROF.) Regions are named based on the habitats defined by Meißner et al. (2014).

Similarity analysis yielded two larger groups of samples, of which one (A) is further divided into two subclusters (Figure 8). The subcluster A1 (65% similarity) contains mostly shallower samples, 169 to 913 m (and one deeper station from 1386 m), from Irminger Basin, Reykjanes Ridge, Iceland Basin and north of the Faroe Islands. These samples are characterised by a high diversity with all 37 amphipod groups recorded. The constant presence (71-100% frequency) of Ampeliscidae, Amphilochidae, Aoridae, Caprellidae, Dulichiidae, Ischyroceridae, Corophiida, Eusiridae, Liljeborgiidae, Lysianassoidea, Oedicerotidae, Pardaliscidae, Phoxocephalidae, Stegocephalidae, and Stenothoidae, is noted here. The shallower group is linked with a small cluster of three deep-water samples (2537-2567 m) collected in the Iceland and Irminger basins having 62% similarity. Compared to the shallower cluster, this group can be defined by the significant absence of two families, the Ischyroceridae and Liljeborgiidae, which were both a constant element in the shallower group.

The second major group, cluster B (Figure 8), contains nine samples at 55% similarity from the Norwegian Sea and east of the Faroe Islands. It is a group of deep-sea samples, 1058 to 2422 m, again aligning with just one shallow sample from 600 m. Twenty-six taxa are found in cluster B, which is characterised by the presence of eight families: Amphilochidae, Calliopiidae, Hyperiopsidae, Lysianassoidea, Oedicerotidae, Pardaliscidae, Phoxocephalidae and Stegocephalidae. Compared to cluster A1, in cluster B, the Ampeliscidae, Dulichiidae, Ischyroceridae, Eusiridae, and Stenothoidae have much lower frequencies (between 11 and 44%).

**Figure 9.**
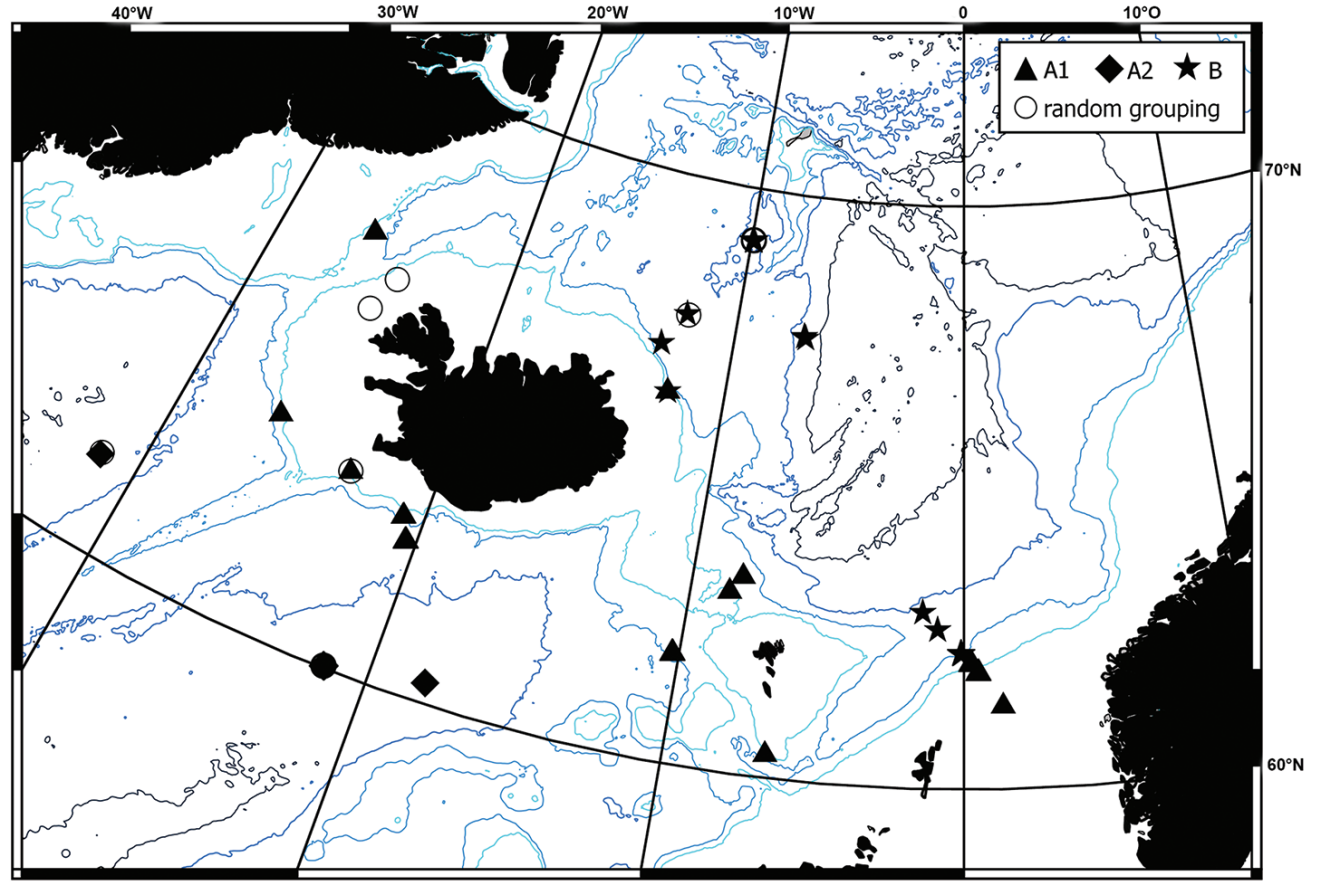
The spatial distribution of the clusters can be associated with the hydrography of the region. Symbols of the clusters from figure 8 are plotted on the station map.

Differences in family richness are also observed between cluster A1 and B. The mean number of families per sample is significantly higher in cluster A1 (Z = 3.951, p=0.00007; 21.4 ± 3.6, min = 17, max = 30) compared to cluster B (11.2 ± 1.8, min = 9, max = 14).

The spatial distribution of the clusters (Figure 9) can be associated with the hydrography of the region. The subcluster A1 consists of very widely distributed samples but their common factor is shallow depths (generally less than 1000 m). A similar pattern was observed for the anthuridean isopod *Calathura brachiata* (Stimpson, 1853) (Negoescu and Svavarsson 1997). This distribution can be linked to the warm surface current (North Atlantic Current) that comes from the south, in the Iceland Basin, divides, with one branch flowing around the Faroe islands, and the second branch encircling Iceland along its south and west coast (Ostmann et al. 2014). The subcluster A2 groups deeper stations from the Iceland and Irminger basins, separated by the Reykjanes Ridge. The lack of a barrier effect has already been observed for other peracarids, including Amphipoda (Svavarsson 1997, Negoescu and Svavarsson 1997, Brix et al. 2014b, Jazdzewska et al. 2018). In the case of subcluster A2 (see Figure 9), the Iceland Scotland Overflow Water, which is a deep-water, cold current moving from north east into the Iceland Basin and later flowing along the Reykjanes Ridge into the Irminger Basin seems to be responsible for shaping the observed assemblage (Ostmann et al. 2014). Finally, the third group recognised consists of deep (middle and deep bathyal) samples from the Norwegian Sea and the east Faroe Islands and may be associated with cold Norwegian Sea deep water (Ostmann et al. 2014). The samples of cluster B have lower diversity in comparison to subcluster A1 and similar differences between Norwegian Sea and northernmost part of North Atlantic Ocean were observed previously for Isopoda (Svavarsson 1997).

### Species level data (Amphilochidae and Oedicerotidae)

Figure 10-14

**Figure 10.**
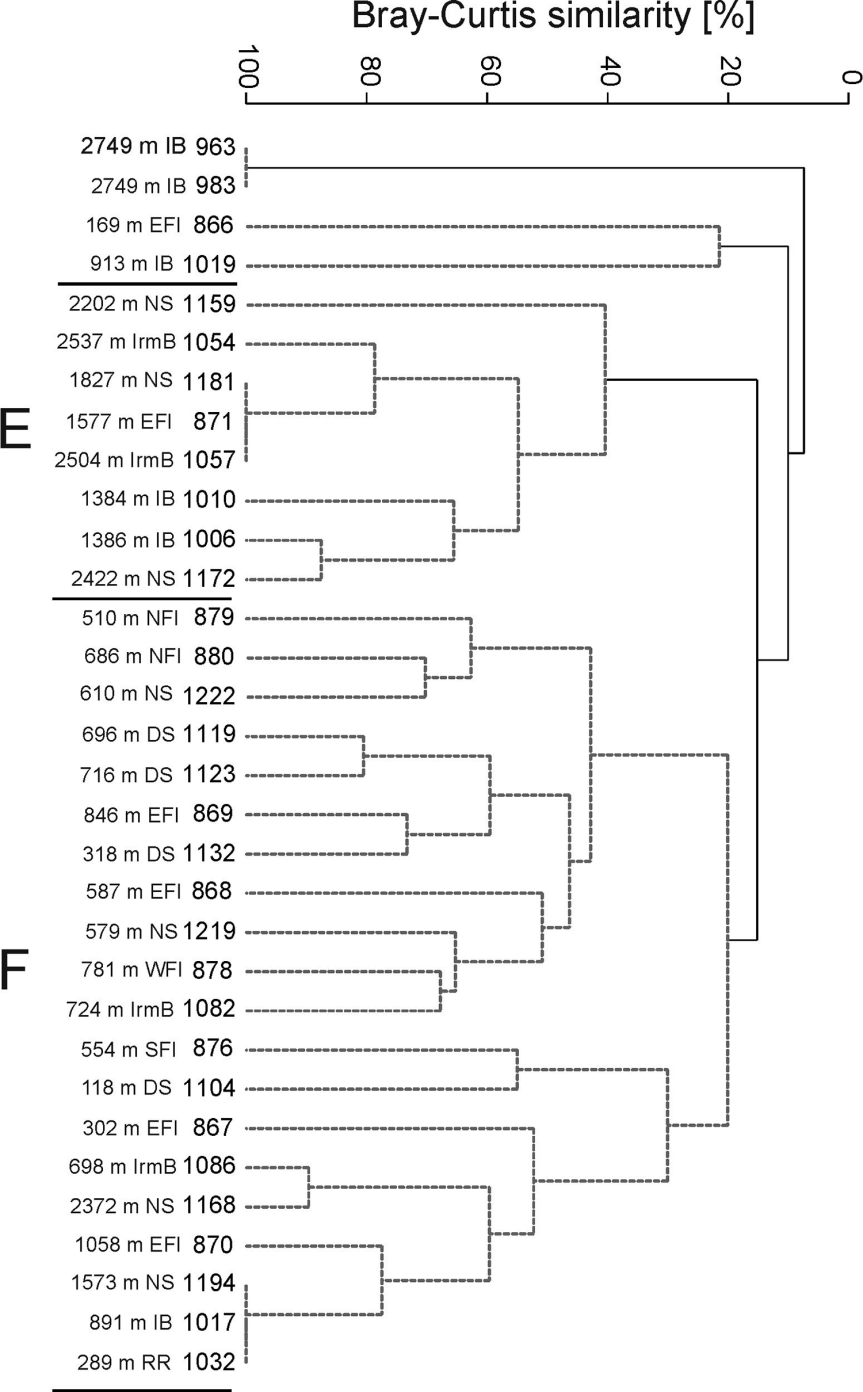
Dendrogram of samples for the Amphilochidae (Bray-Curtis similarity, group average grouping method standardised and square root transformed data). RR - Reykjanes Ridge, NS - Norwegian Sea, DS - Denmark Strait, IB - Iceland Basin, IrmB - Irminger Basin, WFI - west off Faroe Islands, EFI - east off Faroe Islands, SFI - South of Faroe Islands, NFI - North of Faroe Islands. Grey spotted lines indicate the samples that cannot be significantly differentiated by SIMPROF.

For the IceAGE Amphilochidae, similarity analysis demonstrated two larger groups of samples both at relatively low levels of similarity (Figure 10). Amphilochid cluster E, (40% similarity), groups deep-sea samples from different locations (Norwegian Sea, Irminger Basin, and Iceland Basin), and is characterised by a low diversity and dominated by *Amphilochus anoculus* Tandberg & Vader, 2018 (Tandberg and Vader 2018). Amphilochid cluster F (21% similarity) contains mostly shallower samples, 118 to 891 m, in alignment with three deeper samples from 1058 to 2372 m, from various locations. Amphilochid cluster F is dominated by *Amphilochus tenuimanus* Boeck, 1871 and *Amphilochus manudens* Spence Bate, 1862. All 13 species of Amphilochidae were found in the samples forming this cluster.

**Figure 11.**
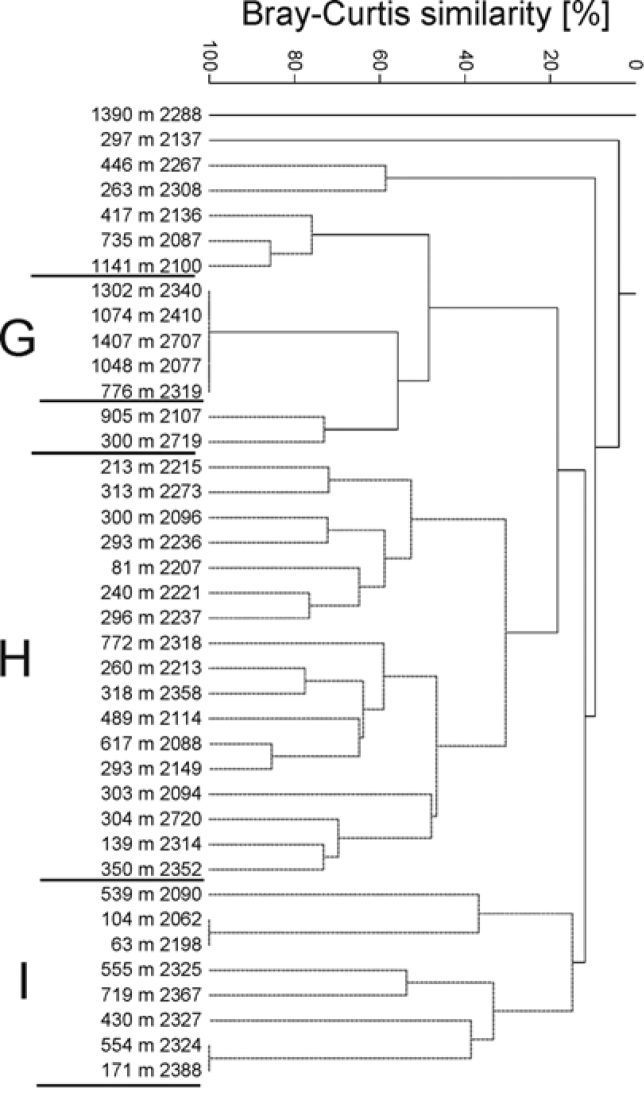
Amphilochidae BIOICE Dendrogram of samples for the Amphilochidae collected during BIOICE project (Bray-Curtis similarity, group average grouping method standardised and square root transformed data). Grey spotted lines indicate the samples that cannot be significantly differentiated by SIMPROF.

In the analysis of Amphilochidae from BIOICE (Figure 11), the similarity analysis yielded two larger clusters at low levels of similarity (cluster H - 30% similarity and cluster I -15% similarity), both containing samples from a similar depth range (from 63 to 772 m) and one smaller group of samples, amphilochid cluster G, collected in a deeper area 1048 to 1407 m and a single sample from 776 m. Amphilochid cluster G (100% similarity), is characterised by the consistent presence of one species *Amphilochopsis hamatus* Stephensen, 1925. Amphilochid cluster H is characterised by high abundance and frequency of *Amphilochus manudens* and *Amphilochus tenuimanus*, while amphilochid cluster I is dominated by *Gitanopsis arctica* Sars, 1892. BIOICE and IceAGE Amphilochidae were analysed in two different datasets due to different abiotic information between the two projects. Overall, the BIOICE amphilochid cluster H corresponds with the IceAGE amphilochid cluster F, while BIOICE amphilochid cluster G is consistent with IceAGE Amphilochidae cluster E (Figure 11), where the depth ranges and common species are the same. Thus, both datasets do show the same pattern.

Although *Gitana* was recorded as a deep and cold-water associated genus in the IceAGE samples, it is known to occur commonly at shallow depths in the North Sea (Beermann and Franke 2011) and the Mediterranean Sea (Krapp-Schickel 1982). However, an affinity with deep and cold water is recognised for the very widely distributed new species *Amphilochus anoculus* (Tandberg and Vader 2018) and *Amphilochopsis hamatus*. *Amphilochus manudens* seems to be limited to the upper 1000 m, with conspicuous abundance at all stations from both BIOICE and IceAGE samples from south-west of the Reykjanes Peninsula (Figure 13). The most abundant amphilochid species, *Amphilochus tenuimanus*, was sampled mainly from shallow waters to the shelf edge (139-905 m, with one record from 1384 m). It is worth highlighting, however, the distinct possibility that *Amphilochus manudens* might be a cryptic species complex (Jazdzewska et al. 2018, Tandberg and Vader 2018).

**Figure 12.**
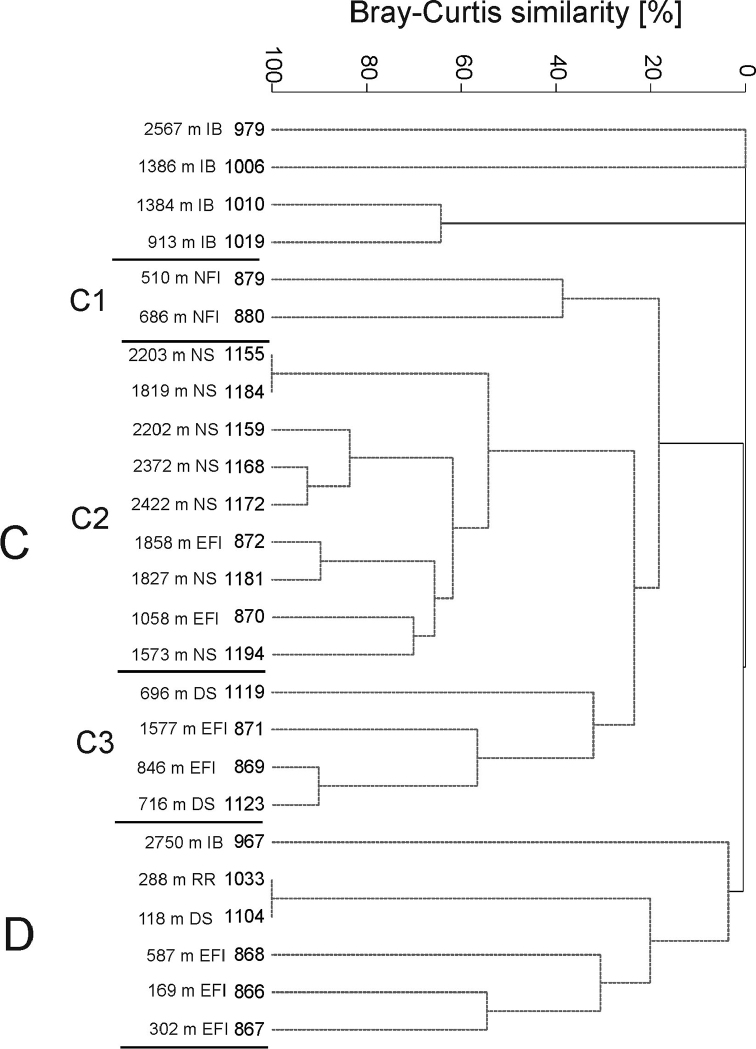
Dendrogram of samples for the Oedicerotidae (Bray-Curtis similarity, group average grouping method standardised and square root transformed data). Abbreviations: RR - Reykjanes Ridge, NS - Norwegian Sea, DS - Denmark Strait, IB - Iceland Basin, IrmB - Irminger Basin, WFI - west off Faroe Islands, EFI - east off Faroe Islands, SFI - South of Faroe Islands, NFI - North of Faroe Islands. Grey spotted lines indicate the samples that cannot be significantly differentiated by SIMPROF.

More detailed analysis of the family Oedicerotidae yields two weakly marked clusters at low levels of similarity (cluster C -19%, cluster D - 4%; Figure 12). Overall, distribution patterns observed for the Oedicerotidae reflected those seen for the higher family-level analysis. In oedicerotid cluster C there is a subcluster of deep-sea samples collected in the Norwegian Sea (C1 - at 62% similarity). This oedicerotid cluster C1 largely corresponds with the family level cluster B. Samples from oedicerotid subcluster C1 are dominated by *Paroediceros curvirostris* (Hansen, 1888), *Deflexilodes tenuirostratus* (Boeck, 1871), *Arrhis phyllonyx* (Sars M, 1858) and *Paroediceros propinquus* (Goes, 1866). Shallower samples are grouped in the oedicerotid cluster D but are also spread across various other subclusters. Oedicerotid cluster D samples are consistently from the continental shelf depths, 118 to 587m, but notably from different regions (Iceland Basin, Reykjanes Ridge, Denmark Strait, north of Faroe Islands). Oedicerotid cluster D is dominated by *Synchelidium haplocheles* (Grube, 1864) and *Monoculodes pallidus* Sars, 1892. This cluster is also typified by the absence of four species which were dominant in oedicerotid cluster C, namely *Paroediceros curvirostris, Deflexilodes tenuirostratus, Arrhis phyllonyx*, and *Paroediceros propinquus.* Eleven of the 21 species of Oedicerotidae have abundances between one and five individuals in the samples.

**Figure 13.**
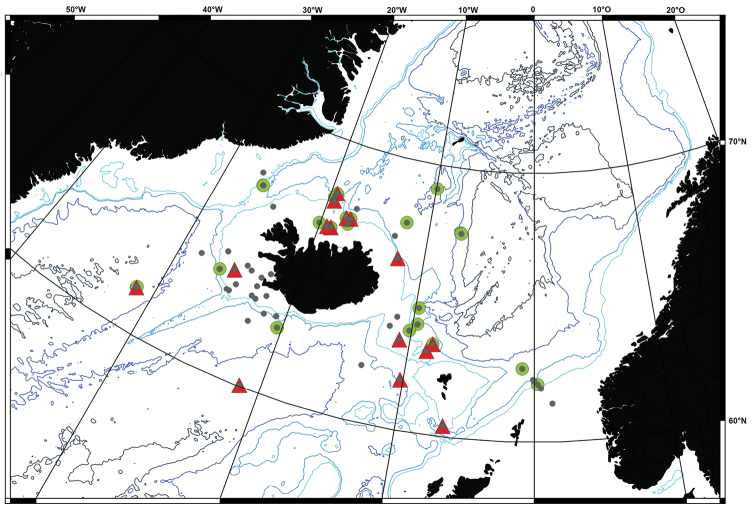
Amphilochidae found at BIOICE and IceAGE stations plotted together (grey circles) with the distribution of *Gitana abyssalis* (red triangle) and *Amphilochus tenuimanus* (green circle) at these stations.

**Figure 14.**
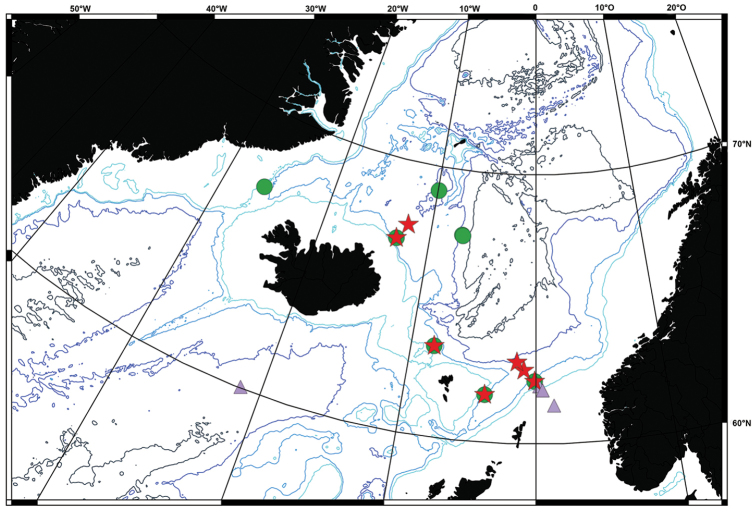
Distribution of selected Oedicerotidae species showing different distribution patterns: *Arrhis phyllonyx* (green circle), *Synchelidium haplocheles* (lilac triangle), *Paroediceros curvirostris* (red star).

In assessing the Oedicerotidae species distribution patterns within the IceAGE material (for examples see Figure 14), a more diverse species assemblage is apparent in the northern sampling localities. The two species of *Westwoodilla*, *W. caecula* (Spence Bate, 1857) and *W. megalops* (Sars, 1883) were present in Norwegian Channel only, as were both species of *Synchelidium*, although *S. intermedium* Sars, 1892 was represented by only eleven specimens across two sites. *Deflexilodes subnudus* (Norman, 1889) (*Monoculodes falcatus*) was present at a single Norwegian Channel site with 17 specimens. *Monoculoides packardi* Boeck, 1871 was recorded at three sites only in the Norwegian Channel, again with one sample represented by a single specimen. A total of 72 specimens were recorded across two regions, the Norwegian Channel and Denmark Strait, for *Deflexilodes tenuirostratus* (Boeck, 1871). A similar split between the Norwegian Channel and Denmark Strait was seen for 217 individuals of *Paroediceros curvirostratus* (Hansen, 1888), yet one of the two Norwegian Channel sites had the majority of individuals, with 110 specimens, while the second site had just three specimens. In the Denmark Strait sites, *P. curvirostratus* specimens were more evenly spread across sites. *Paroediceros propinquus* (Goes, 1866) and *Arrhis phyllonyx* (Sars M., 1858) were present across the Norwegian Channel and Iceland-Faroe Ridge, as well as the Denmark Strait to Norwegian Sea (Figure 14). *Arrhis phyllonyx* also showed high numbers of individuals (42 specimens) at a single Norwegian Channel station, considering a total of 74 specimens reported across all stations.

Oedicerotidae genera and species have the highest diversity in the North Atlantic. As the family has not received intensive study in other regions, with the exception of the north east Pacific, it is difficult to know if this is a biodiverse region for Oedicerotidae or an artefact of taxonomic treatment. Generic level review would be meaningful to address the taxonomic errors in the literature and to better understand relationships where few characters separate groups (Barnard and Karaman 1991). In most genera a full complement of subadult, male, and female specimens have not been assessed, and this is problematic for defining generic level characters, i.e., *Bathymedon* (Just 1980). The distinction between intra- and interspecific variation is not well studied. Some authors accept a 0.1 variation in ratios as an acceptable species-level character even when less than five individuals were available for study and large sample sizes show high degree of overlap and standard error (Jansen 2002). The position of the eye(s) and shape of the rostrum is cited as having high intraspecific variability, e.g., the widely reported *Westwoodilla caecula*, while rostrum shape and size is also used to separate species of *Westwoodilla* (Jansen 2002). The family would benefit from a holistic generic and species level review to more adequately represent evolutionary relationships.

### General findings

The shelf-edge, especially in the Norwegian Channel, is particularly diverse. One possibility for this might be that the two northbound water masses (deep and cold, shallow and warm) mix in this zone, making possible habitats and more abundant food sources for a larger and more diverse set of species. The diversity maximum for gastropod molluscs was found to be between 400 and 450 m (Høisæter 2010). Høisæter's data (2010) for gastropods showed a very similar pattern reported from the Faroe Channel, but with a maximum diversity 200 m deeper along the Norwegian slope close to the Norwegian Channel (North Sea Fan). This zone coincides with a high fluctuation in temperature, where both, positive and negative values, were observed, indicating a varying depth of thermocline. This diversity peak was previously interpreted as an overlap of the upper and lower assemblages (Gage 2004). The factors structuring bathymetric patterns in different ocean basins and slopes may differ from those affecting other taxa in the same area.

While substantial parts of Arctic waters north of Iceland, as well as the North Atlantic south of Iceland, belong to the deep sea, reaching below 3000 m on the abyssal plains, the BIOICE dataset includes only a few stations at these depths. The dataset used here contains several samples below 1000 m. The Icelandic shallow water fauna is well documented, particularly for crustaceans (Sars 1890-1895, Svavarsson 1997, Svavarsson et al. 1993) though information declines with depth. In the case of peracarid crustaceans, in the Arctic Ocean the numbers of individuals of each species is high, while overall diversity is low. Conversely, for the North Atlantic Isopoda, diversity is high, while the number of individuals per species is comparably low (Svavarsson et al. 1993). Svavarsson et al. (1993) demonstrated considerable differences in recent isopod faunal characteristics between the shallow and deep waters North of Iceland. Here, the number of species declines by 50% at 1000 m and to one third of species at depths greater than 2000 m. The degree of Arctic endemism is seen to increase with depth.

Other groups show similar depth patterns. In the case of deep-sea prosobranchs from the North Atlantic (Porcupine Seabight and Abyssal Plain) Olabarria (2006) found depth to be a significant predictor of diversity with rates of species succession increasing rapidly with increasing depth, indicating four possible depths of faunal turnover: 700; 1600; 2800 and 4100 m. The study of Olabarria (2006) was based on data from 71 epibenthic sledge samples between 150 and 4915 m. The turnover depths differed from other taxa in the Porcupine Basin and also from other areas which would indicate a lack of global consistency in such depth-related diversity patterns. The decrease in diversity observed by Olabarria (2006) correlates with the permanent thermocline from about 600 to 1400 m. Rex (1981) and Etter and Rex (1990) found diversity maxima at depths between 2000 to 3000 m for polychaetes, gastropods, protobranchs, and cumaceans. Paterson and Lambshead (1995) found diversity maxima at around 1800 m for polychaetes at the Hebridian Slope. Flach and Bruin (1999) observed increasing diversity with increasing depth in molluscs. On the Scottish Slope macrozoobenthos diversity is low at 400 m and highest at around 1400 m (Bett 2001). The unimodal relationship between diversity and depth with a peak at intermediate depths (2000-3000 m) is not universal and particular abiotic processes can modify the trend (Ramirez-Llodra et al 2010). A recent global biogeography "Global Open Ocean and Deep Seabed" (GOODS) describes 37 benthic provinces divided into four depth ranges (Ramirez-Llodra et al. 2010).

### Conclusions

The sorting effort of two workshops on IceAGE expedition material has enabled the identification of more than 20,000 amphipod specimens to the family level from Icelandic and adjacent waters. Several families were identified further to species level. Distribution maps of occurrences have been provided in a preliminary investigation of regional amphipod family distributions. Statistical analyses at the family level revealed a depth related pattern, which was supported by species level data for two abundant families in the samples, the Amphilochidae and Oedicerotidae. In all three datasets (family level, Oedicerotidae and Amphilochidae) diversity was highest at slope depths where due to upwelling effects, cold water mixes with warmer water and phytoplankton/zooplankton are more abundant, supporting previous hypotheses that thermoclines play an important role in shaping species diversity and distribution patterns in the Icelandic benthic ecosystem (Høisæter 2010).

## Supplementary materials

Supplementary material 1 Table 1. Samples used for present study. Data type: occurence Explanation note: Stations are organised according to the percentage of amphipods identified to the family level. Only the stations marked in green were used for primer analysis, while all were the basis for map preparation. Amphipod taxa are presented from the most to the least abundant in all samples. This is an open access article distributed under the terms of the Creative
Commons Attribution License (CC BY 4.0), which permits unrestricted use, distribution,
and reproduction in any medium, provided the original author and source are
credited.Saskia Brix, Anne-Nina Lorz, Anna M. Jazdzewska, Lauren Hughes, Anne Helene S. Tandberg, Krzysztof Pabis, Bente Stransky, Traudl Krapp-Schickel, Jean-Claude Sorbe, Edward Hendrycks, Wim Vader, Inmaculada Frutos, Tammy Horton, Krzysztof Jazdzewski, Rachael Peart, Jan Beermann, Charles Oliver Coleman, Lene Buhl-Mortensen, Laure Corbari, Charlotte Havermans, Ramiro Tato, Anali Jimenez Campean

Supplementary material 2 Table 2. BIOICE and IceAGE stations used for Primer analysis of Amphilochidae including information about species distribution/number of specimens per station. Data type: occurence This is an open access article distributed under the terms of the Creative
Commons Attribution License (CC BY 4.0), which permits unrestricted use, distribution,
and reproduction in any medium, provided the original author and source are
credited.Saskia Brix, Anne-Nina Lorz, Anna M. Jazdzewska, Lauren Hughes, Anne Helene S. Tandberg, Krzysztof Pabis, Bente Stransky, Traudl Krapp-Schickel, Jean-Claude Sorbe, Edward Hendrycks, Wim Vader, Inmaculada Frutos, Tammy Horton, Krzysztof Jazdzewski, Rachael Peart, Jan Beermann, Charles Oliver Coleman, Lene Buhl-Mortensen, Laure Corbari, Charlotte Havermans, Ramiro Tato, Anali Jimenez Campean

Supplementary material 3 Table 3. IceAGE stations used for Primer analysis of Oedicerotidae including information about species distribution/number of specimens per station. Data type: occurence This is an open access article distributed under the terms of the Creative
Commons Attribution License (CC BY 4.0), which permits unrestricted use, distribution,
and reproduction in any medium, provided the original author and source are
credited.Saskia Brix, Anne-Nina Lorz, Anna M. Jazdzewska, Lauren Hughes, Anne Helene S. Tandberg, Krzysztof Pabis, Bente Stransky, Traudl Krapp-Schickel, Jean-Claude Sorbe, Edward Hendrycks, Wim Vader, Inmaculada Frutos, Tammy Horton, Krzysztof Jazdzewski, Rachael Peart, Jan Beermann, Charles Oliver Coleman, Lene Buhl-Mortensen, Laure Corbari, Charlotte Havermans, Ramiro Tato, Anali Jimenez Campean
